# NetCore: a network propagation approach using node coreness

**DOI:** 10.1093/nar/gkaa639

**Published:** 2020-07-31

**Authors:** Gal Barel, Ralf Herwig

**Affiliations:** Department of Computational Molecular Biology, Max-Planck-Institute for Molecular Genetics, Ihnestrasse 63–73, 14195 Berlin, Germany; Department of Computational Molecular Biology, Max-Planck-Institute for Molecular Genetics, Ihnestrasse 63–73, 14195 Berlin, Germany

## Abstract

We present NetCore, a novel network propagation approach based on node coreness, for phenotype–genotype associations and module identification. NetCore addresses the node degree bias in PPI networks by using node coreness in the random walk with restart procedure, and achieves improved re-ranking of genes after propagation. Furthermore, NetCore implements a semi-supervised approach to identify phenotype-associated network modules, which anchors the identification of novel candidate genes at known genes associated with the phenotype. We evaluated NetCore on gene sets from 11 different GWAS traits and showed improved performance compared to the standard degree-based network propagation using cross-validation. Furthermore, we applied NetCore to identify disease genes and modules for Schizophrenia GWAS data and pan-cancer mutation data. We compared the novel approach to existing network propagation approaches and showed the benefits of using NetCore in comparison to those. We provide an easy-to-use implementation, together with a high confidence PPI network extracted from ConsensusPathDB, which can be applied to various types of genomics data in order to obtain a re-ranking of genes and functionally relevant network modules.

## INTRODUCTION

The analysis of genome-wide molecular data is a complex task and protein–protein interaction (PPI) networks, i.e. the graphical representation of the physical contacts between proteins in a cell, have emerged as a powerful scaffold for integrating different data types and boosting the signal-to-noise ratio of such experiments (1). Network propagation allows combining experimental data with molecular interaction information, such that the topology of the network is used to propagate the data effects throughout the network, and by that amplifying and functionally interpreting the experimental data. This approach covers a wide range of data domains and has been applied, for example, for associating genetic variants with cancer (sub-) phenotypes (2) as well as for deriving patient-specific networks from phosphoproteome analysis (3). Network propagation requires for the data to be summarized such that each gene has a weight that can be used for initializing the propagation process. The process can then be executed, usually in the context of a PPI network, such that after the propagation new weights are obtained for the genes (see Supplementary Methods). These weights can be used to simply re-rank the genes in order to identify novel disease genes, or as an input for a further module identification step, to identify sub-networks which can then be associated with the phenotype under study. Several network propagation approaches have already been used, for example for the identification of novel disease genes (4–6), the discovery of disease-associated network modules (2,7–8) and the prediction of drug-targets (9).

Although network propagation is mathematically rather straightforward, the post-processing of the results is not, and there are still challenges in discovering disease-associated network modules. Some methods use the weights after propagation for re-ranking the genes (10–12). However, in order to identify novel genes that are potentially relevant to the phenotype in question, the problem of selecting the relevant genes based on the new rankings is still present. Some methods select the top genes after the re-ranking (5,12–13), but then a cutoff must be made to decide which genes to further explore. Others implement a module identification step, i.e. they identify sub-networks which contain connected genes, and thus might represent relevant pathways and mechanisms. For example, PRINCE (14) specifically identified modules that represent protein complexes. Other approaches, such as HotNet2 (2) and Hierarchical HotNet (8) apply the same network propagation procedure, but then extract a similarity matrix for every pair of genes in the network. They identify ‘hot’ sub-networks which contain genes with high weights after the propagation, however, calculating a similarity matrix, with a value for every pair of genes, might generate false interactions, as not all genes are directly connected in the PPI network. Finally, there is generally a delicate interplay when trying to identify network modules (15), as on the one hand smaller modules will split functional information that is inherently represented by the interactions, but larger modules might contain false positive genes which are actually irrelevant to the phenotype.

Another challenge for network propagation is the underlying PPI network and its drawbacks. PPI networks are characterized by a power-law degree distribution, where many nodes have a low degree, and only a few an extremely high one (16). The interactions have been experimentally detected, for example with yeast two hybrid systems (17–19). Such systems are very useful in identifying interactions between proteins, however the experimental design may result in a technical bias toward proteins that are used as ‘bait’ when measuring new interactions (20). In addition, these ‘bait’ proteins tend to be more frequently studied, and therefore more interactions involving them are detected (21–23). These experiments result in PPI networks with proteins having an artificially high number of interactions (24) and ‘star’-like substructures with a central highly connected (‘bait’) protein and many less inter-connected (‘prey’) proteins. This creates a problem in the mathematical formulation of network propagation, since the degree of the nodes is used to execute the propagation steps and therefore the propagation will arrive more often to nodes with a high degree. This issue can be addressed by correcting for the degree via some significance test, as previously applied by methods like DADA (25) and RDPN (26). However, a correction for the degree bias has yet to be implemented directly in the mathematical formulation of the network propagation.

In order to address these issues, we first aimed to reduce the bias in network propagation due to the usage of node degree, and then attempted to identify more comprehensive modules based on the propagation results. Instead of degree, we introduce coreness for the mathematical formulation of the propagation. Coreness is a property that can be assigned to each node and reflects whether the node belongs to a very densely connected part of the network or rather its periphery (27). Coreness is a global network property, and can be estimated through a series of steps using the H-index (28), starting from the degree, which is a local property (29). Furthermore, coreness has been shown to reflect how influential a node is in spreading information throughout a network (30). Another benefit of coreness is that central hubs of star-like structures are assigned a low core value (though having a high degree) if their neighbors are not well-connected. Therefore we explored whether coreness could help reducing the negative effects of the degree bias on the propagation process, and thus improving the results. Based on these results we then strived to identify network modules. For that, we proposed to combine the propagation results with a manually curated list of seed genes, and extract sub-networks in a semi-supervised fashion. First we included only the seed genes in a sub-network, and then it was expanded with intermediate nodes according to the propagation results, which we assigned with a significance level using a permutation test. Such approach, to the best of our knowledge, is yet to be applied in combination with network propagation for the identification of network modules.

NetCore's workflow consists of three main steps. As a first step, a PPI network was extracted from ConsensusPathDB (31). This PPI network was recently reported in an independent study as one of the top performing networks for identifying disease genes via network propagation (32). In the second step, summarized genomics data, in the form of gene weights, were propagated in the PPI network, via a mathematical formulation that is implementing node coreness. The propagation allows for a re-ranking of the genes and the identification of genes with significant weights as compared to a network randomization procedure. In the third step, the resulting significant genes, along with a pre-defined list of seed genes, which can be extracted either directly from the experimental data or alternatively from a manually curated database, are further used for identifying network modules. This allowed for detecting modules that are functionally relevant, robust against varying validation gene sets and that expand prior knowledge regarding the mechanisms that are responsible for the phenotype in question. We demonstrated improved performance of NetCore compared to node-degree methods in three different settings: (i) identification of disease genes from the GWAS catalog involving 11 different gene sets, (ii) network module identification for Schizophrenia data and (iii) network module identification and candidate predictions for pan-cancer mutation data. We highlighted important characteristics of NetCore, compared it to three other network propagation methods and showed that it identifies biologically plausible sets of phenotype-associated genes.

## MATERIALS AND METHODS

### Coreness of interaction graphs (k-shell decomposition)

Given a connected graph *G*, we can define the k-core sub-graph *G_k_* as the maximal sub-graph of *G* that contains only nodes with a degree of at least *k*. Clearly for *k = 1*, *G_1_* refers to the entire graph and for every *k_2_* > *k_1_* it holds that ${G_{{k_2}}}$ is a subgraph of ${G_{{k_1}}}.$ The core of a node *v* is then defined by the largest value *k* of the k-core sub-graph that contains *v*.

**Figure 1.**
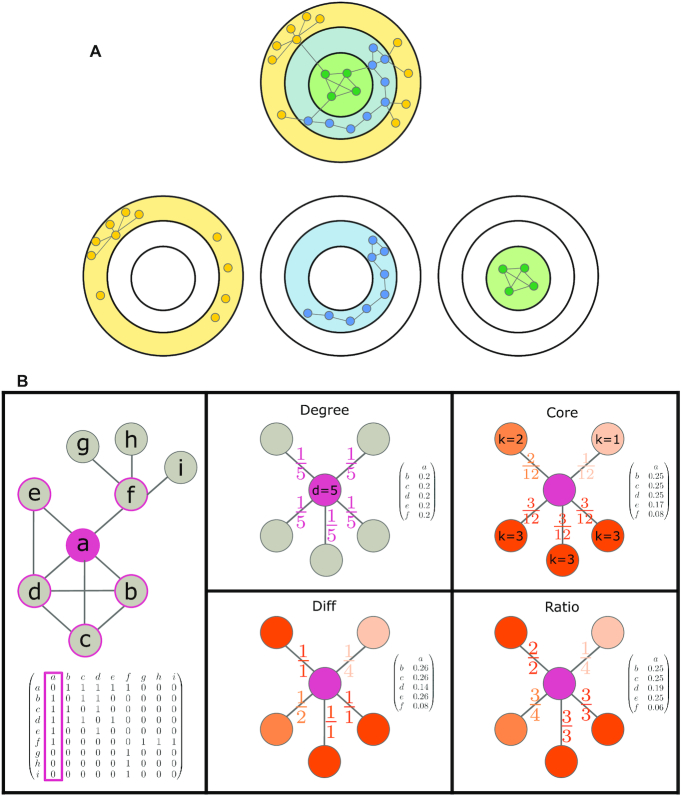
Node core normalization: (**A**) K-shell decomposition: an example network with 25 nodes and 29 edges. In this example, the network is decomposed into three layers, each one representing a different k-core sub-graph. The first layer (k-core = 1) and the nodes in it are colored in yellow, the second in blue (k-core = 2) and the third in green (k-core = 3). (**B**) Adjacency matrix normalization: an example network with 9 nodes and 12 edges with the corresponding adjacency matrix. Node *a* has five neighbors. The normalization methods for the neighbors of *a* are exemplified. When normalizing based on degree only, the probability for walking to any of the neighbors is 0.2, since the degree of *a* is 5. When normalizing based on core only, the probability is according to the core of the neighbor, and normalized by the sum of cores, such that the sum of probabilities is 1. The other two normalizations are based on the difference between the degree and core, or the ratio between them. The vectors show the probability values after normalizing by the sum of differences (or ratios), such that the values sum up to 1.

To compute the core of a node *v* we can apply a k-shell decomposition algorithm (33). The algorithm divides the network into layers (1-shell, 2-shell, 3-shell, etc.) that successively represent the entire network (Figure 1A). The outer layers represent the periphery of the network, while the inner layers with the higher *k* values represent the densely connected core of the network. The algorithm works iteratively: first, all nodes with degree 1 are removed, including their edges. Any remaining nodes with degree 1 after the previous step are then also removed, until none such remain. This results in the first layer of *k*  =  1, and all of the removed nodes belong to it (1-shell). Next, the same procedure is repeated with nodes of degree 2. Those removed nodes will belong to the layer where *k*  =  2 (2-shell). The procedure is continued until it finally arrives at the last layer, where all the nodes will have the highest *k* value in the network.

Coreness can be described as the property of the node to belong to the densely connected part of the graph (higher node cores) or its periphery (lower node cores). Nodes with higher cores are typically referred to as influential nodes since they are able to spread information faster across the network than nodes with lower core values.

### Protein-protein interaction (PPI) network and disease genes data

#### ConsensusPathDB PPI network

In this work we made use of a high confidence PPI network (31) that can be extracted from ConsensusPathDB (release 34). ConsensusPathDB (34) is a meta-database that includes molecular interactions and pathway concepts from 32 different public resources (35). The web interface of the database allows, among others, to search for interactions based on different molecular types, such as genes, proteins, drugs, etc., as well as to conduct gene enrichment and over-representation analysis (36). PPI interactions can be extracted in the form of a network. This network holds more than 300 000 binary interactions and serves as a comprehensive model of the human interactome. Each interaction in the network is scored with a confidence score computed from six different topological and annotation-based indices (37). We have previously shown (31) how to construct a high confidence PPI network by taking only the connected interactions that have a confidence score above 0.95. In this paper we make use of this high confidence PPI network, which holds 10 586 proteins and 114 341 unique interactions. Topological characteristics of this network are described in further details in the Supplementary Methods.

#### GWAS catalog gene sets

We made use of the GWAS (Genome Wide Association Studies) gene sets provided by Huang et al. (32) for testing performance of NetCore. These include nine gene sets associated with a disease, and two gene sets associated with a quantitative trait (Table 1). Each set includes between 100 and 500 genes. The gene sets were extracted from the GWAS catalog (38), which is a large database that provides significant SNP-trait associations. In this catalog, each SNP is mapped to a gene, such that each trait can be associated with a list of genes.

**Table 1.** Gene sets

Source	Disease (Trait)	Gene set size	Coverage in PPI network
GWAS catalog	Body mass index	177	97
	Breast cancer	113	73
	Crohn's disease	436	227
	Height	387	234
	Prostate cancer	324	168
	Rheumatoid arthritis	124	88
	Schizophrenia	395	226
	Systemic lupus erythematosus	137	90
	Type 2 diabetes	124	78
	Ulcerative colitis	345	203
	Vitiligo	111	76
DisGeNet	Schizophrenia	1436	1072
NCG	Cancer consensus	711	626
	Cancer candidate	1661	1050

#### Schizophrenia GWAS data

We applied NetCore to a Schizophrenia genetic variations dataset (39) that was provided by Carlin *et al.* (5). This data-set includes *P*-values for SNP associations to Schizophrenia according to GWAS based on the analysis of 9394 cases and 12 462 controls. Based on the SNPs, genes were assigned *P*-values according to a predefined genomic region of 10 kilobases (kb) up- and downstream of the gene. A gene is assigned the lowest *P*-value from the SNPs that are within this region. This *P*-value is then −log_10_ transformed such that each gene is associated with one weight. A total of 14 966 genes had a weight above 0, but only 10 586 of them were covered in the PPI network and were used as input for NetCore.

For validating the computed Schizophrenia modules we extracted the genes that are associated with the disease according to the DisGeNet database (40). We downloaded the *BeFree* gene-disease associations from the database and extracted all genes relevant for Schizophrenia. This list included 1436 genes, 1072 of them were covered in the PPI network and used for assessing the plausibility of the modules computed with NetCore.

In addition, we evaluated NetCore's performance for identifying Schizophrenia-associated genes using another much larger GWAS dataset by Pardiñas *et al.* (41) (with 40 675 cases and 64 643 controls), which was produced later than the one by the Schizophrenia Psychiatric GWAS Consortium (39). We downloaded the meta-analysis summary statistics and extracted all the available SNPs to *P*-value associations. The SNPs were then associated with genes, according to a predefined genomic region of 10 kb, up- and downstream of it. Each gene was assigned the lowest *P*-value from the SNPs that were identified for its region. We applied a significance level of *P* < 5  ×  10^–8^, which was also used by Pardiñas *et al.* (41), to identify significant SNPs, and remained with 945 significant genes. Out of them, 426 were covered in the PPI network and were used for the evaluation of NetCore.

#### Cancer mutation data

We applied NetCore to a pan-cancer somatic mutation data set (42). The dataset includes a total of 4742 samples from 21 tumor types, 12 of them from The Cancer Genome Atlas (TCGA) and 14 from non-TCGA projects at the Broad Institute. The mutations from all samples were combined together, such that duplicated patients and duplicated mutations were removed. For each tumor type a total of 18 388 genes were analyzed, and three significance metrics were calculated using the following methods: MutSigCV (43), MutSigCL and MutSigFN (44). MutSigCV calculates for a gene the number of non-silent mutations, and the *P*-values are determined on a background model that is based on the number of silent mutations in the surroundings of the gene. MutSigCL measures the significance of the positional clustering of the observed mutations, while MutSigFN measures the evolutionary conservation in the positions of the mutations. Both measures are assigned a *P*-value based on a permutation test for the non-silent coding mutations. Finally, the three metrics were combined into a single *P*-value, which was then corrected for multiple testing into a single FDR Q-value using the Benjamini and Hochberg method. This resulted in 1489 genes with a *Q*-value < 1, which we then −log_10_ transformed and scored with in order to initialize the node weights prior to network propagation. For evaluating NetCore's results, we made use of The Network of Cancer Genes (NCG) catalog (45). The catalog contains manually curated information from publications about more than 2000 cancer-associated genes, which are known or predicted to have driver roles in cancer based on somatic mutations. These genes are divided into two categories: (i) 711 ‘*cancer consensus*’ genes, which include both tumor suppressors and oncogenes and (ii) 1661 ‘*cancer candidate*’ genes, genes that were identified by mutational screenings and have strong support to be involved in cancer development. We used both lists to evaluate network propagation predictions for the somatic mutation data.

All data resources are listed in Table 2.

**Table 2.** Resources and softwares

Resource/Software	Link	Citation
NetCore	https://github.molgen.mpg.de/barel/NetCore	This paper
ConsensusPathDB PPI Network (version 32)	http://cpdb.molgen.mpg.de/download/ConsensusPathDB_human_PPI.gz	(34)
GWAS gene sets	https://github.com/idekerlab/Network_Evaluation_Tools/	(32)
Schizophrenia GWAS Consortium	http://nbgwas.ucsd.edu/nagadata/	(5)
Schizophrenia GWAS Pardiñas *et al.*	https://walters.psycm.cf.ac.uk/	(41)
Cancer MutSig Q-values	http://www.lagelab.org/wp-content/uploads/2017/06/NetSig_Code.zip	(42)
NAGA	https://github.com/shfong/naga	(5)
HotNet2	https://github.com/raphael-group/hotnet2	(2)
Hierarchical HotNet	https://github.com/raphael-group/hierarchical-hotnet	(8)
Network of Cancer Genes (NCG) (version 6.0)	http://ncg.kcl.ac.uk/index.php	(45)

### Network propagation with NetCore

Network propagation is a general name for several mathematically equivalent formulations which allow executing a diffusion process over a network. Three main formulations have previously been described (1): the first is a diffusion kernel, which is also referred to as heat kernel; the second is a random walk, which is commonly used for electric networks with a specific source and target; the third is a modified version of a random walk called random walk with restart (RWR) which is also known as insulated heat diffusion or personalized PageRank (46). This version allows for controlling the trade-off between prior information and network smoothing.

#### Random walk with restart—mathematical formulation

For NetCore we chose to use the RWR formulation. This formulation includes three main components: (i) the interaction network, (ii) a scoring scheme for each node in the network and (iii) a restart parameter α. Given an interaction network G, we can extract the adjacency (adj.) matrix A, which can then be normalized into another matrix W (further details in the following section). The input weights for the network nodes are represented with a vector p_0_, and at every step k the weight vector p_k_ is calculated according to (Equation 1).

(1) $$\begin{equation*}{{{p}}_{{k}}} = {{\alpha }}{{{p}}_0} + (1 - \alpha ){{W}}{{{p}}_{{{k}} - 1}}\end{equation*}$$

This process has been shown to converge to a steady-state with final propagation weight vector *p* (Equation 2) when two conditions are matched: (i) the interaction network is connected, (ii) the normalized adj. matrix W is stochastic (i.e. the eigenvalues are at most 1 in absolute value). The high-confidence PPI network from ConsensusPathDB consists of a big connected component with 10 586 nodes and 114 341 edges. This network is used for applying RWR (see further details in the Supplementary Methods).

(2) $$\begin{equation*}{{p = }}\alpha {({{I}} - (1 - \alpha ){{W}})^{ - 1}}{{{p}}_0}\end{equation*}$$

#### Adjacency matrix normalization

The most common normalization of the adj. matrix A is by using the node degree (Equation 3), where D is a diagonal matrix with the node degrees.

(3) $$\begin{equation*}{{W}} = {{A}}{{{D}}^{ - 1}}\end{equation*}$$

In addition to the common degree normalization, we applied three other normalizations that involve the core of the nodes (Figure 1B). The first is based only on core. Given the adj. matrix A and the core values of all the nodes in the network *K* = (k_1_…k_n_) we normalize each column using the core values of the neighbors of the node associated with that column. Thus, for each neighbor we divide its core value by the sum of core values of all neighbors (for an example, see Figure 1B). Therefore, densely connected neighbors of a node will gain more weight than nodes that are in the periphery of the graph. This results in a normalized version of the adj. matrix *A*^core^, which is a stochastic matrix, as the sum of every column is always 1. The definition of the matrix is given by (Equation 4).

(4) $$\begin{equation*}{A}_{i,j}^{{\rm core}} = \frac{{{k_i}}}{{\mathop \sum \nolimits_{l|Alj \ne 0} {k_l}}}\ \end{equation*}$$

The second normalization is based on the difference between the node degree and node core. Since there is a bias in the degree of some nodes in the network, we wanted to correct for it using the core. A node with a high degree, that is due to study bias, will be connected to many other nodes, which are themselves not well studied and therefore do not have many connections in the network. Consequently, due to the nature of the k-shell decomposition, these nodes will belong to a lower k-core layer, and therefore the node with the high degree will then have a low core value. Thus, a large difference between the degree and the core suggests to a study bias, which we then aim to correct by penalizing for this difference. In order to do that, we defined the normalization such that each neighbor's value is normalized according to this difference. After each column is normalized as such, we applied a further normalization and divided each column by its own sum, so that the normalized adj. matrix *A*^diff^ is stochastic. The definition of the matrix is given by (Equation 5).

(5) $$\begin{equation*}A_{i,j}^{diff} = \frac{1}{{\left( {{d_i} - {k_i}} \right) + 1}}\ \end{equation*}$$

The last normalization is similar to the previous one, except it is using the ratio between the degree and core, rather than the difference. The reasoning is similar, we wanted to penalize for nodes with a very high degree yet a rather low core. Here, for each node, we normalized directly using the ratio between its core value *k* and its degree *d*. Due to the nature of the k-shell decomposition algorithm, the core can never be larger than the degree, and therefore the ratio will always be equal or smaller than one. The smaller the ratio, the bigger the degree and the lower the core. We then further normalized the adj. matrix and divided each column by its own sum, such that the normalized adj. matrix *A*^ratio^ is stochastic. The definition of the matrix is given by (Equation 6).

(6) $$\begin{equation*}A_{i,j}^{{\rm ratio}} = \frac{{{k_i}}}{{{d_i}}}\ \end{equation*}$$

#### Restart probability parameter

The restart parameter α defines the probability of the random walk to restart again. This allows to control how much of the input weights will be diffused throughout the network. The lower the value, the less the walk restarts and therefore more of the weight is spread in the entire network. Thus, given a PPI network, the parameter can be set once, regardless of the initialization of the weights. To calibrate the value for the ConsensusPathDB PPI network, we compared between three cases: low value (*α* = 0.3), intermediate value (*α* = 0.5) and high value (*α* = 0.8). We tested the performance of NetCore for these three values for identifying GWAS gene sets, and examined how many of the reported genes in NetCore are present in the GWAS gene sets (see Supplementary Figure S1). In five of the 11 GWAS gene sets the number of reported genes that are in the input gene set is the highest for *α* = 0.8, in four of the gene sets the number is highest with *α* = 0.5 and in two with *α* = 0.3. We note that the value of α allows to control the trade-off between finding novel disease-associated genes and including potential false predictions. We therefore decided to set *α* = 0.8 as default in order to be able to still predict novel disease genes, while reducing the number of potentially false predictions. However, for other PPI networks this parameter can be modified by the user.

### NetCore evaluation

#### Statistical significance of node re-ranking with NetCore

NetCore identifies significantly re-ranked nodes using two parameters: the final propagation weights and *P*-values derived from network randomizations. In order to assign a significance level to NetCore's re-ranking results we applied normalization with random degree-preserving networks (RDPN) (26). This method is based on randomizations of the input network, such that the propagation weight of each node is compared to the propagation weights obtained using randomized degree-preserving networks. To generate such networks we used the double-edge swap algorithm, as implemented in the Python software Networkx (47). The algorithm allows to execute at most n random swaps by randomly choosing at each step 2 edges (u, v) and (x, y), removing them and creating the new edges (u, y) and (x, v), unless they already exist. The swaps are kept only if the network after the swap stays connected. Once n such random networks are generated, the significance level is calculated using the propagation weights achieved with these random networks. Thus, the *P*-value p_v_ for node v, with its propagation weight w^(v)^, for n random networks and corresponding propagation weights $w_1^{( v )}, \ldots ,{\rm{\ }}w_n^{( v )}$, is defined in (Equation 7). In our analyses we generated *n* = 100 random networks so that the minimal *P*-value that can be achieved is *P* = 0.0099. This threshold is chosen as default in our analysis but for other set ups this can be changed by the user.

(7) $$\begin{equation*}{p_v} = \frac{{\left| {\left\{ {i|w_i^{\left( v \right)} \ge {w^{\left( v \right)}}\ \forall i \in \left( {1, \ldots ,n} \right)} \right\}} \right| + 1}}{{n + 1}}\ \end{equation*}$$

#### Evaluation of normalization methods

We evaluated the performance of the four different normalization methods in a 5-fold cross validation scheme using the 11 GWAS gene sets from the GWAS catalog (Table 1) and the ConsensusPathDB PPI network as scaffold. Our goal was to test, given a set of input disease genes, which method can predict best novel disease genes. Thus, given a pre-defined list of genes that are known to be associated with a disease, we sub-sampled it five times into a validation and a training set, such that the size proportion was 1:4 respectively. Each training set was then used to execute the network propagation four times, one for each normalization method. All the genes in the training sets were given an initial weight of 1, to reflect their disease association and the rest (in the entire network) of 0. The weights after the propagation were assigned *P*-values, as described in the previous section. If a gene achieved a significant enough *P*-value, then it is predicted to be a novel disease association. To assess the sensitivity of the predictions, given a range of significance levels (*P*-values range from 0.01 to 1), all the genes in the network were sorted according to their *P*-values and the receiver operating characteristic (ROC) curve was calculated, according to the validation and training sets. Hence, if a gene was associated with a *P*-value below the significant one, and it was present in the validation set, then this gene is referred to as a true positive prediction. However, if it is not present in the validation set, then it is a false positive prediction. For the ROC curve calculations, a negative set was defined by randomly choosing nodes from the network that match the degree of the nodes in the training set, since the number of nodes in the training set is much lower than the number of nodes in the rest of the network. The five ROC curves were than averaged into one ROC curve to get a consensus curve, from which the area under the ROC (AUROC) was calculated.

### Module identification in NetCore

We established a novel semi-supervised approach for identifying modules based on network propagation results in combination with prior knowledge. We aimed to identify sub-networks that are biologically relevant for a given condition, and therefore combined prior knowledge about genes (seed genes) together with the propagation results. Given a set of seed genes, our implementation allows for propagation from these, using a binary scoring scheme as input and identifies modules based on the results. Additionally, we also provide the option to apply the propagation step using experimental data, as long as they can be summarized into one weight for all or some nodes in the network. The propagation is then applied to the input weights, and the identification of modules can be based on the propagation results together with the prior knowledge about the genes that are relevant for the data. If such prior knowledge is not available, the modules can also be identified based on the input weights and the propagation weights only. In such cases, we generate the seed list gene according to the input weights and use the top 100 genes for the module identification step.

The following steps are applied in order to identify modules in NetCore (further details are given in the next section):

- Extract seed-induced sub-network (i.e. the seed genes and their interconnecting edges)
- Extend seed-induced sub-network with nodes for which the following three conditions hold:
  - Significant weight after propagation (*P* < 0.01)
  - Direct neighbor of at least one seed node
  - High enough weight after propagation (w > w_min_)
- Separate extended seed sub-network into modules by identifying connected components.

#### Extension of seed modules using *P*-values and propagation weights

Given a set of seed genes, we first extracted the sub-network that includes only these genes (nodes) and their connections (edges) (Step i.). We term this a seed-induced sub-network. In case no seed genes are available, we extracted the sub-network based on the original input weights. We ranked the genes by their input weights, and used the top 100 genes for extracting the seed sub-network. Then, after applying network propagation, we used the results to add more genes into the seed-induced sub-network. We set a threshold for a significant *P*-value *P* < 0.01 of the propagation weights, and considered only the genes with a *P*-value below this threshold (Step ii. a). We used 0.01 as we apply a permutation test with 100 random networks, and therefore this accounts for the minimum level of significance we could achieve. These genes are ranked according to their weights after the propagation. Finally, from this ranked list, we added genes that have at least one connection to one of the genes in the seed sub-network (Step ii. b), such that their weight is above a minimum threshold. The weights after the propagation depend mostly on the input weights, and the restart parameter, which controls how much of the input weight is spread in the network. Therefore, we decided to set the minimum weight threshold based on the distribution of the weights after the propagation. This minimum weight threshold is set to be the 75th percentile of the propagation weights of the significant nodes that are not already in the sub-network (Step ii. c). Finally, we search for the connected components in the extended seed-subnetwork and output each one as a network module (Step iii.).

#### Evaluation of NetCore's modules connectedness via entropy

In order to evaluate the connectivity of the identified modules, we measured the entropy of the seed nodes distribution with respect to the modules and compared it to the maximum possible entropy. Since the modules are the connected components of the extended seed sub-network, we could calculate the entropy of the seed nodes contained in these *M* modules by: E = $ - \mathop \sum \limits_{{M_i}} {p_{{M_i}}}\log {p_{{M_i}}}$ such that ${p_{{M_i}}} = \frac{{{k_{{M_i}}}}}{n}$ where, ${k_{{M_i}}}$ is the number of seed nodes in module ${{\rm{M}}_{\rm{i}}}$ and n is the total number of seed nodes. Each seed node that is not covered by one of the computed modules is assigned a ‘module’ of size 1. The maximum entropy, calculated by ${E_{{\rm max}}} = \log n$, reflects the case where all seed nodes are in different modules. Therefore, we measured the connectedness as the difference between *E*_max_ and E, which reflects the distance from the maximum entropy. The larger the distance, the more seed nodes are inter-connected in the same modules and the nodes are less distributed over a large number of smaller modules. The same calculation can also be applied to the modules within the seed sub-network, where the modules are the connected components in the sub-network. Thus, the connectedness of the seed sub-networks and extended seed sub-networks can be directly compared.

#### Evaluation of NetCore's modules via over-representation analysis

Over-representation analysis allows to identify if a computed set of genes (i.e. a network module) is statistically significantly enriched in another pre-defined gene set (for example, a pathway), given a background list of all genes. The statistical significance is obtained via a hypergeometric test, where a *P*-value is calculated based on the number of identifiers that are present in the computed module and in the pre-defined pathway gene set. This type of analysis is available via the ConsensusPathDB web server. ConsensusPathDB includes a total of 5436 pathway gene sets from 12 different resources: Pharmgkb, Ehmn, Humancyc, Wikipathways, Inoh, Netpath, Reactome, Signalink, Kegg, Biocarta, Smpdb, Pid. In this work, we applied over-representation analysis via ConsensusPathDB to the genes from the different modules that were identified by NetCore. We used a minimum module size of 10 genes. Furthermore, we used all the genes from the high confidence PPI network in ConsensusPathDB as background, and extracted only the significantly enriched pathways. A minimum overlap of two genes between the input list and the pathway gene list was required. Only pathways with a *P*-value of 0.01 were extracted, and we used the *Q*-values, which are corrected for multiple testing, as a measure for the level of enrichment.

#### Other network propagation methods

We compared NetCore with other existing methods that also apply network propagation for module identification (Table 2). We focused on three such methods: NAGA (5), HotNet2 (2) and Hierarchical HotNet (8). In NAGA the authors recommend to re-rank the genes after the propagation and take the top 100 genes along with the induced subnetwork. Then they use ModuLand (48), a network clustering method implemented in Cytoscape, to compute clusters, which serve as the final identified modules. HotNet2 and Hierarchical HotNet are both based on the same network propagation formulation, but apply a different approach for extracting the modules. Both methods define a similarity matrix S based on the random walk with restart calculation. HotNet2 then builds a fully connected graph from S, removes edges below a minimum threshold δ, and extracts the strongly connected components (SCC) from the graph, which then serve as the final modules. The output consists of four values for δ, which is estimated from the data and the results, and so we compared only to the results extracted from the minimal δ value because this value yielded the largest final modules. Hierarchical HotNet constructs a hierarchy of clusters from S consisting of SCCs, estimates the optimal cut for the hierarchy, and the generated clusters are reported back as modules. Of these modules, we only compared to those that consisted of at least two genes. All network propagation methods were applied to the ConsensusPathDB PPI network, with a restart parameter of 0.8, in order to allow fair comparisons.

### NetCore implementation

NetCore is implemented in Python3 (releases 3.6 and 3.7) and is available via github: https://github.molgen.mpg.de/barel/NetCore. NetCore is built on the Networkx (47) package for manipulation of complex networks. The computation of the RWR formula, and the calculation of the steady state distribution (Equation 2), is implemented using the linear algebra module of the SciPy software for Python (49). The running time for one such computation (on a Linux machine with 64 cores) takes 30 s. Since NetCore implements a permutation test that is based on random networks, the total running time depends on the number of permutations. Given 100 permutations, each one requires a computation of 30 s, and therefore a total of 50 min. The overall running time, including the module identification, requires no more than 60 min. In addition, to execute NetCore's permutation test, we provided an implementation for generating random degree-preserving networks. The running time for creating such networks depends both on the size of the network (number of edges) and a constant factor, which controls the number of attempts to swap edges. For the ConsensusPathDB network (114 341 edges) and a swap factor of 100, generating one random network takes up to 45 min. Since multiple networks can be generated at the same time, we provided a fast implementation that runs in a parallel fashion using Python's multiprocessing module. To generate 100 random networks, using 64 cores, a total running time of 90 min is required. The computation of the random networks needs to be executed only once for every input network, and can later be used repeatedly for running NetCore.

## RESULTS

### NetCore - a network propagation workflow for identifying disease modules using coreness

**Figure 2.**
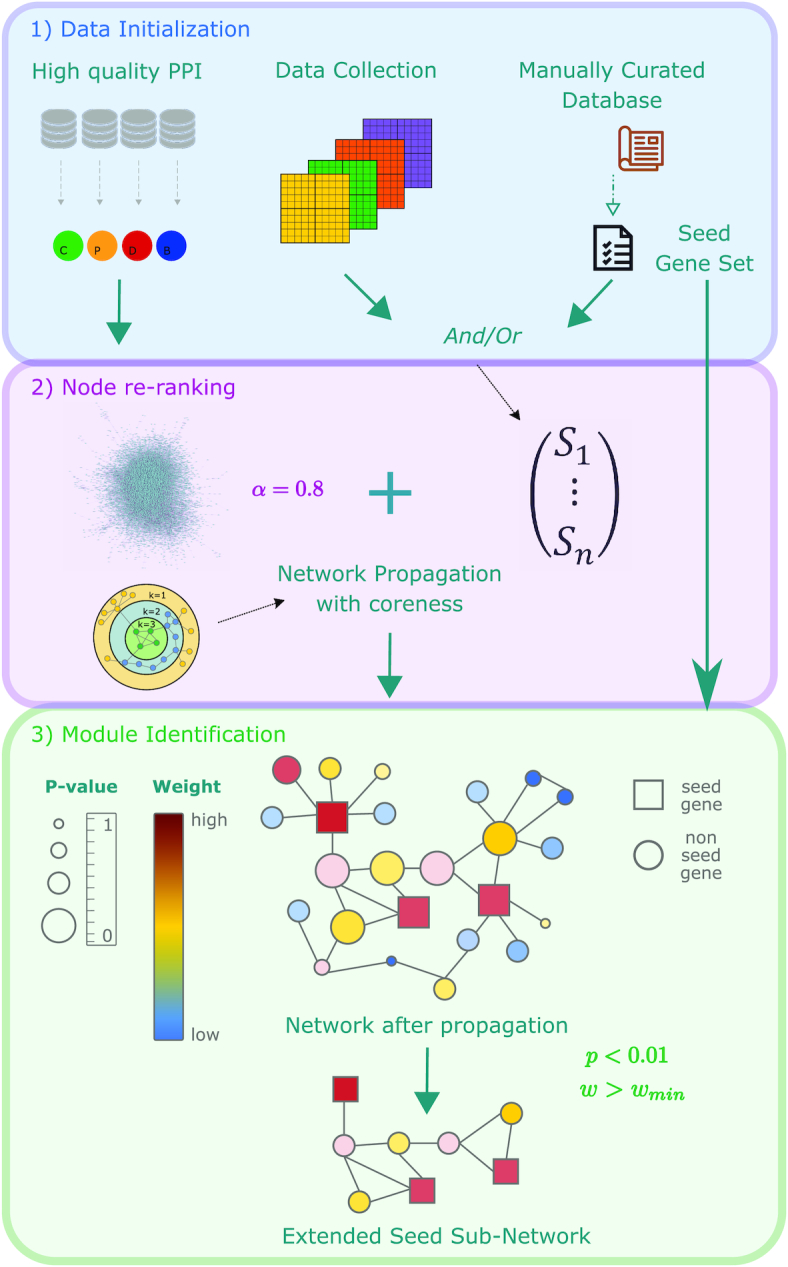
NetCore workflow: (1) Data initialization, which includes the extraction of a scaffold PPI network, experimental data and extraction of a seed gene list representing *a priori* knowledge. (2) Network propagation using node coreness which involves initialization of node weights with experimental data or alternatively with a weighted list of seed genes, random walk with restart propagation specifying the restart parameter α (default *α* = 0.8), and assigning a propagated final weight and a *P*-value (through permutation analysis) to each node. (3) Module identification in a semi-supervised fashion combining both network propagation results and the seed gene list. The seed genes are connected by PPIs and neighbor nodes are added that have a significant *P*-value and a sufficient weight after re-ranking.

The NetCore workflow consists of three main steps (Figure 2): (1) data initialization, (2) node re-ranking and (3) module identification. (1) The first step includes the extraction of a PPI network, which we obtained from the ConsensusPathDB database (see ‘Materials and Methods’ section and Supplementary Methods). In addition, experimental data need to be summarized into weights, such that for each gene, *i*, an input weight, *S_i_*, is computed that reflects its experimental outcome. Alternatively, a list of seed genes, extracted from a manually curated database, may also be used as input, allowing for example to apply a binary scoring scheme. These genes can also later be used as seed genes for module identification. (2) In the next step network propagation based on a random walk with restart (see ‘Materials and Methods’ section) is applied, such that a normalization step based on node coreness is implemented (see ‘Materials and Methods’ section), and a final re-ranking of the nodes is obtained, along with a significance assignment based on degree-preserving randomized networks. For the propagation, the restart parameter α needs to be set (default *α* = 0.8; see ‘Materials and Methods’ section). (3) In the last step, gene modules are identified in a semi-supervised way based on the propagation results together with the seed gene list. The module generation starts with the nodes corresponding to the seed genes and their connecting edges (seed sub-network). Next, intermediate nodes are added according to their *P*-values and weights after the propagation (extended seed sub-network). Here a significance level is set according to the number of permutations, and a minimal weight is computed directly from the underlying data. We used a *P*-value of *P* < 0.01 and set the minimum weight w_min_ according to the distribution of the weights after re-ranking (see ‘Materials and Methods’ section). Our approach is implemented in Python3 and is available via https://github.molgen.mpg.de/barel/NetCore.

### Coreness normalization reduces PPI network bias and improves over degree normalization in network propagation using random walk with restart (RWR)

**Figure 3.**
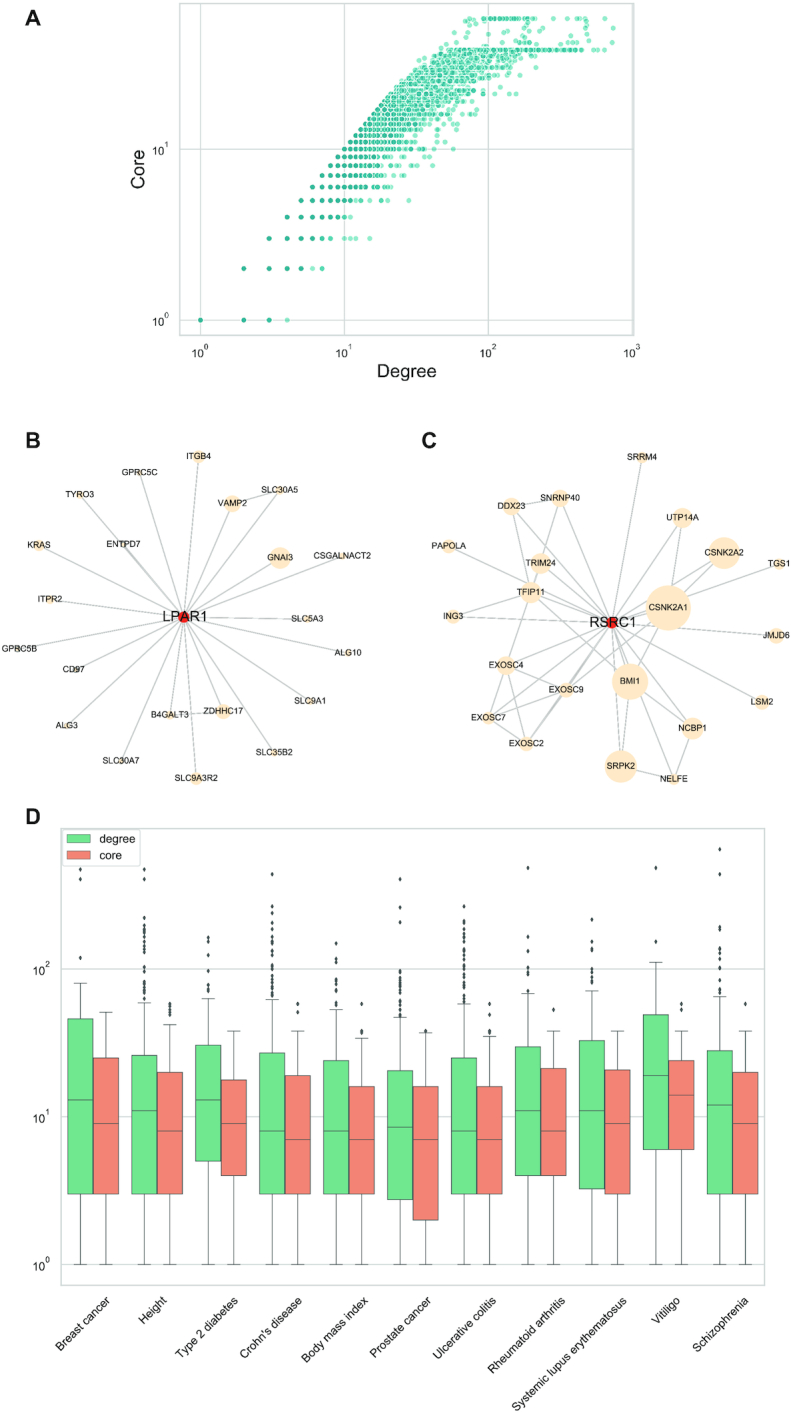
Node degree and coreness in the high confidence ConsensusPathDB PPI network: (**A**) Interdependency of degree (*X*-axis) and core (*Y*-axis) of nodes in the PPI network. (**B**) The neighborhood of *LPAR1* (degree = 21, core = 8) and (**C**) of RSRC1 (degree = 21, core = 18) in the PPI network. The two genes have the same degree but different core values, indicating that *RSRC1* is located in a more densely connected part of the graph than *LPAR1*. This can be seen from the neighbor nodes since *RSRC1* is connected to more nodes which have a lot of interactions, whereas neighbors of *LPAR1* have rather low numbers of interacting partners. Node sizes of neighbors are displayed proportional to their degree. (**D**) Box plots of degree (green) and core values (orange) for the genes in 11 GWAS gene sets. *X*-axis denotes the phenotypic traits and diseases.

In contrast to degree which is a local property of the nodes, we introduced coreness as a global property in the random walk with restart formulation (see ‘Materials and Methods’ section). Figure 3A shows the relation between degree and core for all the nodes in the PPI network. By definition the core of a node cannot be higher than its degree and typically is much smaller. The plot has a typical ‘boomerang’ shape, and while there is a positive correlation, nodes with the same core can still vary an order of magnitude in their degree. This indicates again toward a degree bias, with nodes which have a very high degree but a lower core value (see Supplementary Methods). These ‘hub’ nodes are also very often associated with diseases, as already shown previously for cancer (24). In particular, the core is an indicator whether the node is located in a less densely connected region of the network, even when the degree is considerably high. For example, *LPAR1* (*Lysophosphatidic Acid Receptor 1*), which is associated with *Height* according to GWAS studies, has a degree of 21 but a core of 8 only. Figure 3B shows the neighborhood of *LPAR1* in the PPI network. Out of its 21 neighbors, 16 have a degree that is lower or equal to 21. The rest have a degree of between 27 and 83. In contrast, Figure 3C shows the neighborhood of *RSRC1* (*Arginine And Serine Rich Coiled-Coil 1*), which is also associated with *Height*, and has the same degree of 21, but a core of 18. *RSRC1* is connected to nodes with a much higher degree, all but one have a degree of at least 21, and up to 382. Thus, the core reflects that this gene is located in a much denser region of the network than the previous node. We further explored the degree and core distributions of the 11 GWAS sets (Table 1) and observed that for all gene sets the variation of core values is much smaller than that of the degree (Figure 3D). The coefficient of variation (CV) for all the genes in the GWAS sets is twice higher for the degree than the core (CV_degree_ = 1.9, CV_core_ = 0.9). Additionally, we note that the degree distributions include many extreme outliers, which is not the case for the core distributions. Indeed, there are some genes with a high degree that are present in many of the GWAS sets. For example, *HLA-B* (*Major Histocompatibility Complex, Class I, B*), which has a degree of 135 and core of 35, is present in six of the 11 sets.

**Figure 4.**
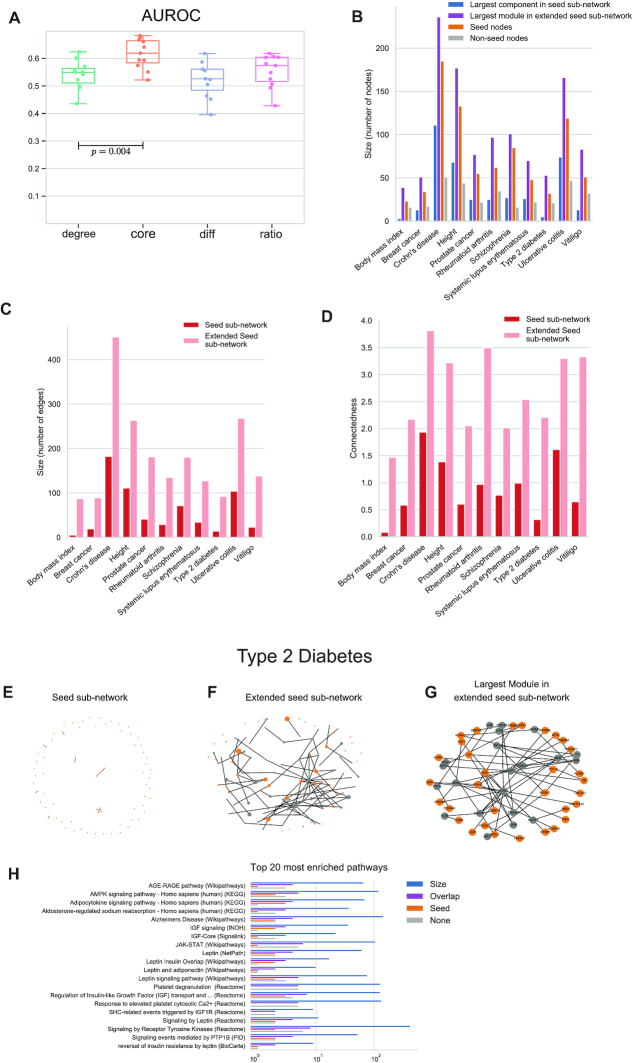
GWAS gene sets results: (A) AUROC for 11 GWAS gene sets using different normalization schemes for the random walk with restart matrix normalization. Degree = standard normalization based on node degree; core = normalization based on coreness; diff = normalization based on difference between degree and coreness; ratio = normalization based on ratio between coreness and degree. *P*-value was computed with a paired Wilcoxon test using the AUROC values of degree and core. (**B**) Number of nodes in semi-supervised module identification for each GWAS data set: (i) the largest connected component for the sub-network containing the seed nodes (in blue), (ii) the largest module using the re-ranking results after network propagation with core normalization (in purple), (iii) the number of seed nodes in the largest module (orange), (iv) the number of non-seed nodes in the largest module (in gray). (**C**) The number of edges in the seed sub-network and in the extended seed sub-network. (**D**) Connectedness of modules in seed sub-network and of modules in extended sub-network after network propagation, measured with an entropy criterion. Bars show the connectedness, which is measured by the difference in entropy from the maximum entropy and the respective modules. (**E**–**G**) Modules for Type-2 diabetes. Orange nodes show genes in the original seed list, and gray nodes show significant genes that were added after network propagation. Graphs show: seed subnetwork (E), extended seed subnetwork (F) and the largest module of the extended seed subnetwork (G). In (F) the size of the nodes is proportional to the weight after propagation. (**H**) The most enriched pathways for the genes in the largest predicted module (G). In blue is the size of the entire pathway, and in purple is the overlap of the genes with the largest module. Orange indicates genes are from the seed list, and gray not.

In order to demonstrate the impact of core normalization on the RWR network propagation we have applied the method to the problem of inferring disease genes. We made use of the node coreness definition to normalize the adjacency matrix in different ways and compared the standard degree normalization with three other normalization schemes using: core only, the difference between core and degree, and the ratio between core and degree (see ‘Materials and Methods’ section). We applied the different normalizations to the 11 GWAS gene sets (Table 1) in a 5-fold cross validation scheme (see ‘Materials and Methods’ section) where we used 80% of the genes as training set and 20% of the genes as validation set for each trait. We used a binary node initialization scoring scheme, where the genes in the training set are scored with 1, and the rest of the genes in the PPI network with 0 and then computed the performance on the validation set after propagation. For each gene set, we calculated the average ROC curve (Supplementary Figure S2) and then the AUROC. Figure 4A shows box plots of the AUROC values for all 11 GWAS gene sets using the different normalization schemes. It can be seen that for most of the gene sets, the core normalization achieves the highest AUROC. On average, there is a significant improvement when using core- instead of degree-normalization in the RWR network propagation (Wilcoxon signed-rank test, *P* =  0.004).

### NetCore's seed-based approach generates highly connected modules with GWAS disease genes

After applying RWR network propagation with core normalization, we sought to identify network modules based on the propagation results. To enforce biological guiding of the network propagation through incorporation of prior knowledge, we applied a semi-supervised approach for module identification. Thus, we used the genes from the GWAS sets as seed nodes, and extended the sub-networks that are induced by them with significant genes after network propagation. First, we extracted the sub-network that connects the seed genes only (seed sub-network) (Supplementary Figure S3). We note that for most GWAS sets, the majority of the genes are not directly connected to each other in the PPI, and the biggest connected component in most cases consists of a few genes only. Then, we extended the seed sub-networks by adding neighbors of seed nodes, based on the propagation results. Namely, we only added neighbors if their weight after the propagation was larger than w_min_, and their *P*-value was significant (*P* < *0.01*). w_min_ is computed from the data and defined for each GWAS set separately according to the distribution of the weights after the propagation (see ‘Materials and Methods’ section). The effect of *w_min_* on the size of the resulting extended seed sub-network is shown in Supplementary Figure S4. We added the new nodes such that we also added all of their respective connections to seed nodes in the sub-network (see Supplementary Figure S5). This resulted in larger connected components which included more of the original seed nodes, and therefore represent a more comprehensive network module (see Supplementary Figure S6). For all 11 GWAS sets, the number of nodes (Figure 4B), as well as the number of edges (Figure 4C), increased in the final modules in comparison to the largest components in the original seed sub-networks, by factors of 2.2–10.6 (nodes) and 2.4–17.4 (edges). More seed nodes were included in the final module, and additionally newly predicted genes were added as well. As a numerical indicator for the connectedness among the seed nodes, we computed an entropy criterion (see ‘Materials and Methods’ section) derived based on the seed gene content of the different connected components of the sub-networks and subtracted it from the maximum entropy, where each seed node is in its own component. Thus, the higher the difference is, the less sparse the network is, and the seed genes are better connected. Using this entropy difference as a measure of connectedness, Figure 4D shows that the connectedness of the seed nodes is always much higher for the extended seed sub-network (factors ranging from 2.0 to 17.86).

We exemplify the results for Type-2 diabetes genes from the GWAS catalog in Figures 4E-H. The seed sub-network (Figure 4E), includes 78 seed nodes but only 14 edges. Most nodes are not connected to each other, and the connected components are rather small, in sizes that range between two and five (connectedness of 0.3). Clearly, it is difficult to extract a functional module that is relevant to the disease based on this sub-network alone. Therefore after propagation we added to the sub-network intermediate nodes which are connected to seed nodes in the PPI network, depending on their propagation results (*P* < 0.01, w > 0.015). This resulted in an addition of 39 nodes and 78 edges to the sub-network (Figure 4F), which then consisted of 15 connected components, in sizes between two and 53 (connectedness of 2.2). The largest connected component, shown in Figure 4G, consisted of 53 nodes, out of which 32 were from the original GWAS set and 21 were not, with a total of 64 edges. Far more seed genes are now interconnected, which corresponds to a 6.9 higher level of connectedness; in addition the seed genes are interconnected to other genes that serve as novel predictions for the disease. We evaluated the functional relevance of this module via an over-representation analysis (see ‘Materials and Methods’ section). Figure 4H shows the top 20 most enriched pathways (*Q*-value < 0.012) for the module, and for each pathway the number of nodes from the module that are part of this pathway, and whether they were included in the original seed list or not. In many relevant pathways the amount of novel candidates is even higher than the amount of original seed genes. Some of the identified novel predicted genes participate in more than one of the most enriched pathways. For example: *IGF1*, *IGF2* and *LEPR*, which have previously been associated with diabetes (50–53), while *STAT5B* and *ROCK1* have shown weaker associations to the disease (54–58). *STAT5B* and *ROCK1* are members of the JAk/STAT pathway, which has recently been shown to influence processes relevant for obesity and diabetes (59).

### NetCore improves consistency among different GWAS studies and identifies novel disease gene candidates—application to Schizophrenia

In order to compute disease relevant network modules we applied NetCore to a genetic variation Schizophrenia study (39) and converted the GWAS-derived *P*-values of the genes to input weights (see ‘Materials and Methods’ section). We compared NetCore with a network propagation method specifically designed for GWAS data analysis called NAGA (Network Assisted Genomic Association) (5). We applied NAGA, which is based on the degree-normalized RWR model, to the same input weights and performed network propagation on the ConsensusPathDB PPI network. Then, as suggested by Carlin *et al.*, we extracted the top 100 genes according to the re-ranking after propagation. To allow direct comparison of the methods we extracted also the top 100 genes according to NetCore propagation results.

**Figure 5.**
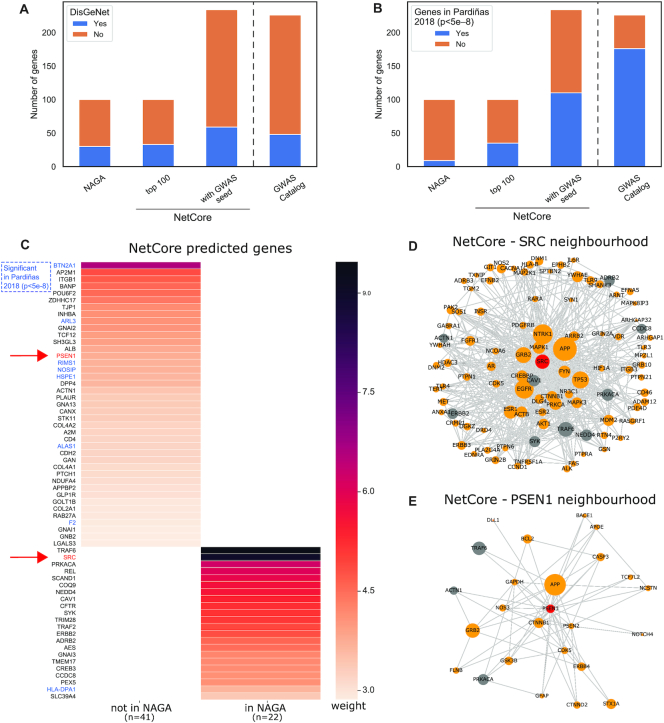
Schizophrenia GWAS-based network propagation: (**A**) The number of genes, and if they are contained in the DisGeNet Schizophrenia disease gene list (blue) or not (orange), for the following (left to right): NAGA network propagation top 100 genes; top 100 genes computed with NetCore; NetCore predicted genes with the 221 GWAS-derived genes (Table 1) as seed list. The last bar shows the overlap of the 221 GWAS-derived genes with the DisGeNet genes. (**B**) The same analysis results as in (A) but with the overlap computed with the genes that were significant (*P* < 5 × 10^–8^) in a recent Schizophrenia GWAS study by Pardiñas *et al.* (**C**) Sixty-three novel candidates predicted from NetCore, using the Schizophrenia-associated genes from DisGeNet as seed nodes either overlapping with NAGA (22 genes) or not (41 genes). The color of the heatmap indicates the weight after the NetCore propagation. Genes that are marked in blue were significant (*P* < 5 × 10^–8^) in the GWAS study by Pardiñas *et al.* The neighborhoods of (**D**) SRC and (**E**) PSEN1 in NetCore's largest module. Orange genes are in the DisGeNet list, gray are novel predictions. The sizes of the nodes are proportional to the weights after propagation.

In order to highlight the advantages of including prior knowledge for the module identification step, we also applied NetCore using 226 genes from the GWAS catalog (Table 1) as seed nodes and computed an extended seed sub-network from these genes along with the propagation results. We evaluated the results by calculating the overlap to Schizophrenia-associated genes derived from the DisGeNet database (40). Although the DisGeNet gene list is not independent of the list from the GWAS catalog, this comparison demonstrates the power of incorporating prior information in NetCore. The performance (measured by overlap with DisGeNet) was improved when the genes from the GWAS catalogs were used as seed nodes. Figure 5A shows the overlap between the different gene lists and the 1,072 Schizophrenia-associated genes in DisGeNet. NAGA’s top 100 genes have an overlap of 30, while NetCore's top 100 genes overlap with 33, an increase of 10% in comparison to NAGA. The list generated using the 226 GWAS seed nodes increases the overlap further to 59 genes. Interestingly, the GWAS seed set itself includes only 48 genes from DisGeNet, i.e. when using NetCore propagation with the seed genes from GWAS, we gained 11 more disease-associated genes from DisGeNet and, thus, improved the consistency of the two datasets.

In order to further test consistency of network propagation among disease gene sets we evaluated the different network propagation outcomes with a second set of genes that were found to be significant (GWAS *P*-value < 5e^−8^) in a larger and more recent Schizophrenia GWAS study (41) (see ‘Materials and Methods’ section). Whereas NAGA’s top 100 genes overlap with only nine of those significant genes, NetCore's top 100 genes overlap with 35 of them. Hence, NetCore appears robust with respect to different validation sets with respect to the same disease (Figure 5B).

In order to predict novel Schizophrenia candidate genes we applied NetCore's propagation and module identification again, this time using the genes from DisGeNet as seed genes, since this is the most comprehensive a priori gene list (1072 genes). With these seed genes NetCore identifies 1136 genes in the extended seed sub-network where the largest module consists of 951 genes. Of these 888 genes are shared with DisGeNet, which is to be expected, as they were used as seed genes. 63 genes are potential ‘novel’ disease genes, 22 of them are also predicted by NAGA’s top 100 approach. Figure 5C lists the 63 novel candidates grouped according to whether they were also predicted by NAGA, and ranked according to their weight after propagation with NetCore. We notice that the genes with the highest weights after the propagation, for example *TRAF6* and *SRC*, are also predicted by NAGA. This is to be expected, as NAGA takes the top 100 ranked genes after propagation. But, there are also some genes with intermediate weights, which are only predicted by NetCore, such as *BTN2A1* and *AP2M1*. *BTN2A1* was also found significant in the more recent Schizophrenia GWAS study (‘Materials and Methods’ section), in addition to seven more genes that were predicted by NetCore.

We further explored two of the predicted novel associations: *SRC* and *PSEN1*. We show their neighborhood within the computed module in NetCore in Figure 5D and E. *SRC*, has 97 neighbors, 87 of them from DisGeNet (*a priori* seed; orange) and 10 are novel predictions (gray), including *SRC* itself. The sub-network is fairly dense, with 548 interactions, 97 alone belong to *SRC*. Other highly connected genes in the sub-network are: *EGFR*, *GRB2* and *ESR1*, all of which are already associated with Schizophrenia according to DisGeNet. Among the newly predicted genes are *TRAF6* and *PRKACA*, which are ranked first and third respectively after propagation, and also appear in the NAGA predictions (Figure 5C). Both of these genes had a rather small initial weight based on the data, and a substantial increase in their weight after the propagation. *PSEN1* has 24 neighbors and is connected to other well-known disease genes, such as *APP* and *GRB2*. *PSEN1* encodes a presenilin protein, which is associated with other neurodegenerative diseases, in particular Alzheimer's disease. Mutations in *PSEN1* have been identified as one of the first genes related to early onset Alzheimer (60).

### NetCore identifies novel cancer candidate genes with implications to patient survival

**Figure 6.**
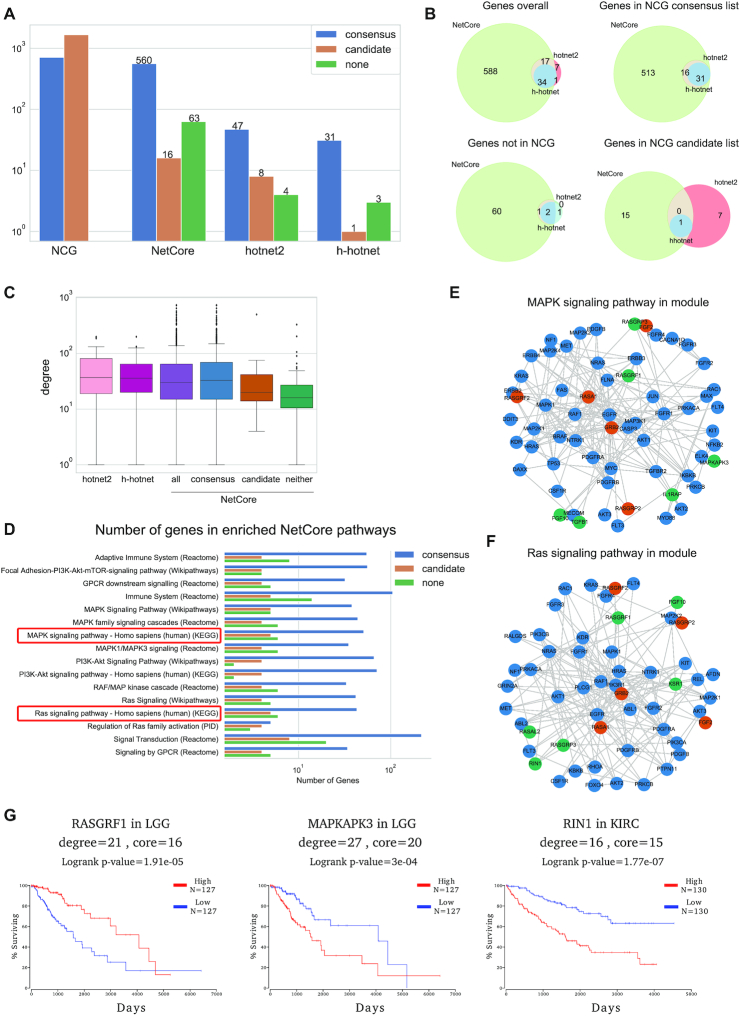
Network propagation analysis with pan-cancer mutations: (**A**) The number of genes in the predicted modules reported by NetCore, HotNet2 and Hierarchical HotNet (h-hotnet). Colors indicate the different categories of the NCG lists (blue = cancer consensus genes, orange = cancer candidate genes, green = genes in neither lists). (**B**) Venn diagrams showing the overlap between the three methods for all the genes in the computed modules, and according to the different NCG categories. (**C**) Box plots of the node degrees of the computed module genes. For NetCore this is also broken down to the different NCG categories. (**D**) Pathways with at least three predicted genes from the candidate list in the biggest module (633 genes) from NetCore. Color indicates the different NCG categories. (**E**) The genes from the biggest module that are part of the MAPK signaling pathway according to KEGG. (**F**) The genes from the biggest module that are part of the RAS signaling pathways according to KEGG. Blue genes are in the consensus cancer list, orange in the candidate and green are new predictions. (**G**) Cox regression plots for three of the novel predicted genes from the modules in (E) and (F) generated using OncoLnc (62). LGG refers to Brain Lower Grade Glioma. KIRC refers to Kidney renal clear cell carcinoma.

We applied NetCore to pan-cancer mutation data from 21 tumor types (‘Materials and Methods’ section). As input weights, we used MutSig *Q*-values (42), which summarize the significance of the mutational frequency, clustering and functional impact of the mutations in all genes. As seed nodes for the module identification, we used the consensus cancer gene list (‘Materials and Methods’ section) from the Network of Cancer Genes (NCG) repository (45). This repository holds genes identified from cancer sequencing screens with strong (‘consensus cancer genes’), as well as less strong (‘candidate cancer genes’), driver evidence. For comparison with NetCore, we also applied on the same data two other network propagation approaches that were developed for the analysis of cancer mutation data: HotNet2 (2) and Hierarchical HotNet (h-HotNet) (8). Both methods make use of degree-based RWR network propagation, but apply a different module identification approach, and have been previously used to identify significantly mutated sub-networks in pan-cancer mutation data. We extracted the modules from the three methods and compared them based on their genes. Figure 6A shows the number of genes in the modules, and their overlap with the genes from the NCG cancer consensus and candidate lists. Both HotNet2 and h-HotNet have a relatively small number of genes in their modules. In all methods, the majority of the genes belong to the consensus list. This is of course to be expected from NetCore, as we used the consensus genes as seed for retrieving the final modules. However, NetCore also retrieves more candidate genes due to its semi-supervised approach. This is further shown in Figure 6B, where NetCore's genes almost completely overlap with the other two methods, but include 15 more genes from the candidate list. HotNet2 reported seven genes from the candidate list, but those are not overlapping with the 15 genes reported by NetCore. We explored the degree of the nodes in all the modules (Figure 6C), and observed a higher degree in both the HotNet2 and h-HotNet modules. This is due to HotNet2 and h-HotNet mainly reporting genes from the consensus list, and hardly reporting any genes with a lower degree. We divided the genes from NetCore's module according to the NCG lists, and noticed a lower degree both for the genes from the candidate list, as well as the genes that are in neither lists. This shows NetCore is less biased by node degree and is able to predict also genes that have a lower degree than those in the consensus list.

We further evaluated the cancer-association potential of the novel predicted cancer genes that are neither consensus nor candidate genes via an over-representation analysis (see ‘Materials and Methods’ section). We applied the analysis to the genes in the NetCore modules and extracted the most enriched pathways (*Q*-value < 0.01). We then investigated those enriched pathways that contained at least three genes from the candidate list (Figure 6D; *Q*-value < 2.7e-6). All pathways also included genes that are non-candidate genes, where some pathways include more genes from the candidate list, and others don’t. We focused on two KEGG (61) pathways: ‘*MAPK signaling pathway*’ (*Q*-value = 1.11e-19) and ‘*Ras signaling pathway*’ (*Q*-value = 2.05e-20), and show the sub-networks from NetCore's module that include the genes that are enriched for these pathways (Figures 6E and F). The majority of genes are in the consensus list (blue), while a smaller number of genes are in the candidate list (orange) or in neither of the two lists (green). In total, both pathways include nine genes that are in neither of the NCG lists: *RASGRP3*, *FGF10* and *RASGRF1* are present in both pathways; *RASAL2*, *KSR1* and *RIN1* in ‘*Ras signaling pathway*’ only; and *IL1RAP*, *MAPKAPK3* and *TGFB1* in ‘*MAPK signaling pathway*’ only.

We argued that these novel genes might still be cancer-relevant biomarkers since they are connected with many consensus and candidate genes. We thus examined the associations of these genes to cancer survival data by generating Cox regression plots using OncoLnc (62) and displayed the results for those cancer types with the lowest FDR-corrected *P*-values. In Figure 6G we show the results for three of the novel predicted genes from the modules, which were part of the *MAPK* and *Ras* signaling pathways: *RASGRF1*, *MAPKAPK3* and *RIN1*. All three genes are significantly associated with survival of cancer patients, and therefore could potentially be used as biomarkers. These genes have a rather low degree, exemplifying the power of NetCore's semi-supervised approach in identifying novel biomarkers. In addition, we repeated the same analysis for genes in the overview ‘*Pathways in Cancer*’ list from KEGG that were predicted in NetCore's module but are in neither of the NCG lists. This is the most enriched pathway in the module (*Q*-value = 1.34e-65). It consists of 475 genes, 147 of them are in NetCore's module. From those, 137 are from the consensus list, three from the candidates list, and seven are in neither lists. Again, we identified six of the seven novel genes as potential biomarkers due to their significant correlation to patient survival data: *CTBP1*, *FGF10*, *LPAR1*, *LRP5*, *RASGRF3* and *TGFB1* (Supplementary Figure S7).

## DISCUSSION

We developed NetCore, a network propagation method that uses node coreness instead of degree in the mathematical implementation of a random walk with restart. The novel method accounts for the fact that experimental bias and study bias of PPI networks result in a high node degree bias in PPIs (24). Contrary to degree, node core is a global property (29) that suggests to the level of influence a node has in spreading information in a network (30). We showed the relation between node degree and core in the ConsensusPathDB PPI network, and how it can reduce the high degree bias for the identification of GWAS gene sets. Degree bias in PPI networks have been previously addressed in the context of network propagation, but was only applied to adjust for the statistical significance of the results (25,26). We therefore decided to address the bias by directly adjusting the adjacency matrix, before applying the propagation. There exists other modifications to the random walk with restart, such as random walk with extended restart (63), where each node has its own restart probability, however such an adjustment of the adjacency matrix has yet to be introduced when applying network propagation. We established three different normalization schemes for the adjacency matrix that are based on core, and compared their performance in the context of network propagation for identifying GWAS gene sets. We concluded that core normalization is performing significantly better than degree and therefore recommend using it when executing random walk with restart-based network propagation.

The methods proposed for identifying network modules after applying network propagation still suffer from various drawbacks. They either simply extracted the genes with the highest weight after the propagation (5), or those with a significant *P*-value after applying a permutation test (26), but those are not necessarily connected such that a comprehensive module can be extracted. Other methods focused on identifying modules that represent protein complexes (64), but of course modules can include many more proteins and represent various processes, like signaling pathways. Finally, both HotNet2 (2) and Hierarchical HotNet (8), which apply different module identification schemes to network propagation results, can potentially include false interactions between proteins, that don’t exist in the PPI network. We therefore addressed the module identification problem in a semi-supervised way. We proposed to first extract a list of knowledge based seed genes, and then extend it according to the network propagation results. Our approach introduces several improvements in comparison to others. First, we exploited prior knowledge to determine an initial set of genes that should certainly be present in the desired modules. Second, we applied a permutation test in order to calculate a significance level for the weights after the propagation, such that we utilized both the weight and the significance when identifying novel genes. And finally, we identified modules by first extracting a seed sub-network, which was then extended with non-seed genes, such that all relevant interactions from the PPI network were also included. By that, we were able to identify modules that were functionally relevant to the phenotype, were enriched in various pathways and included novel predictions for genes that could be involved in the underlying mechanisms of action.

We first applied NetCore to 11 GWAS gene sets and identified network modules that included both the input genes and novel candidate genes that are potentially relevant to the phenotype. By adding intermediate nodes, we were able to both connect between seed genes, that didn’t have a direct connection in the PPI network, and predict novel genes that are involved in the same pathways as the seed genes. We showed that all of NetCore's modules were larger and contained more seed nodes, when comparing to the components of the sub-network that is induced by the seed nodes only. We also quantified the connectedness of the resulting modules using an entropy measurement, which showed that NetCore's modules are less dispersed and more inter-connected. We exemplified this with Type 2 diabetes, which had a very low number of connections between the genes associated with the disease in the PPI network. We could substantially extend the seed sub-network with more genes, which (i) connected between many seed genes and (ii) had a significant weight after the propagation. The largest module was enriched with pathways related to *leptin*, which has been linked to diabetes before (65–67), as well as some *IGF* (*Insulin Like Growth Factor*) related pathways. These pathways included both genes that were already associated with the disease, as well as some novel candidates. Among those were *IGF1* and *IGF2*, which already have stronger associations to diabetes (51,52). Additional genes include members of the JAK/STAT signaling pathway, e.g. *STAT5B* or *ROCK1*, where it has been argued that this pathway is dysregulated in metabolic diseases including obesity and diabetes (59).

We further demonstrated the advantages of NetCore for identifying novel disease genes and modules for Schizophrenia. By combining experimental evidence from a large genetic variation study, and a list of well-known disease genes extracted from a curated database, we were able to predict novel candidate genes that might be associated with the disease. While some of these results are shared by another method, others are only reported by NetCore. Furthermore, we demonstrated NetCore's relevance by showing that it can predict novel genes that are significant in future GWAS studies. For example, *BTN2A1*, which is the highest scoring gene reported only by NetCore, would be a promising candidate for further studies. It has already been associated with other disorders such as dyslipidemia (68–70). We identified *SRC*, a tyrosine-protein kinase, which is connected to many genes that are known to be associated with Schizophrenia, such as *APP*, *MAPK1* and *NTRK1*. Dysregulated *SRC* has been previously linked to Schizophrenia together with the activity of *NMDA* (71–74). *GRIN2A* and *GRIN2B*, which are some of the subunits of *NMDA*, are also connected to *SRC* in NetCore's module. In addition, we identified another candidate gene, *PSEN1*, which is also connected to *APP* and other well-known disease genes, but is not a neighbor of *SRC*, and thus might be involved in a different mechanism that affects the disease. *PSEN1* has already been implicated in Alzheimer's disease (75–78) as well as other neurodegenerative and neuropsychiatric disorders (79). *TRAF6*, *PRKACA* and *ACTN1* are all connected to both *SRC* and *PSEN1* and therefore it might be useful to further investigate their interactions in order to better understand the mechanisms that could be involved in Schizophrenia.

We also tested NetCore's ability to predict novel cancer genes based on cancer mutation data and compared the results to the state-of-the-art methods in the field, HotNet2 and Hierarchical HotNet. Both methods apply a similar network propagation approach, but implement a different module identification procedure. They have previously been shown to predict the highest numbers of candidate cancer genes (8), in comparison to other network based methods for cancer gene predictions (6,80–81). However, we have shown that both methods, while being very specific, reported a rather low number of novel cancer genes, and mainly those with a high node degree. We showed that NetCore, being a semi-supervised approach, can detect more candidate cancer genes, which have a lower degree, and are also connected to well-known consensus cancer genes. This indicates yet again the power of (i) using core instead of degree for network propagation and (ii) using a pre-defined list of seed nodes in order to predict novel genes that can be associated with the disease. This is especially relevant for cancer, as it is extensively studied, and therefore there are multiple sources from which a comprehensive seed list can be extracted. Finally, we showed that we were also able to provide novel predictions that have yet to be implicated in cancer. We focused on genes from the *MAPK* and *Ras* signaling pathways, which are well known to be involved in cancer development, and estimated their potential relevance as biomarkers via their ability to predict patient survival, based on gene expression data, with respect to available patient cohorts. *RIN1* expression has already been implicated in tumor development and invasion (82–84). Hypermethylation of *RASGRF1*, has been suggested as a biomarker for colorectal cancer (85). However, while *MAPKAPK3* has already been suggested as a potential biomarker for colorectal cancer (86) further investigations are still in order.

NetCore runs with three essential parameters, the restart probability $\alpha$, as well as the *P*-value and minimal weight thresholds, that determine the significant genes after the network propagation process and those that will be added to construct the final network modules. It should be emphasized that while $\alpha$ has been adjusted for the ConsensusPathDB PPI network from GWAS data (default $\alpha \ = \ 0.8)$, the other parameters are computed from the data itself after the propagation process and, thus were not previously optimized. Furthermore, $\alpha$ has to be adjusted only once, as it depends only on the PPI network and not on the input data. It is in principle possible though to tune the final modules with an additional post-processing step that includes, for example, the core value of the genes. If one wishes, for example, to further exclude genes from the periphery, and focus on the densely connected genes, one could remove genes with a low core value. For instance, if we were to apply a threshold of core > 5 for NetCore's results on pan-cancer mutation data, this would only exclude one of the genes that are already in NCG’s candidate list, and reduce the detected novel candidates from 63 to 57 (and by that also the potential false positives), while preserving the potential survival biomarkers shown in Figure 6G.

We have demonstrated how NetCore can improve network propagation results for the identification of novel disease genes and modules. However, in addition to the modifications that we have proposed, there are still several challenges to address and room for advancement. First, the node degree bias in PPI networks can be further reduced by designing more accurate experiments for studying PPIs, without focusing on well-characterized nodes only. In addition, while ConsensusPathDB is already integrating over multiple resources (34,35), it has been shown that summarizing information from multiple PPI networks can even further improve the results (32). Second, for an ideal performance, NetCore requires a set of seed genes in order to identify comprehensive modules. Great efforts have been made in the field of cancer for comprising lists of genes that are associated with the disease (45,87–88), and it would be very beneficial to produce similar curated lists for other diseases too, in order to improve the module identification process and identify novel candidates via NetCore. Third, network propagation can further be used as a method for integrating multiple OMICs data (1). For example, it has been applied for the identification of cancer genes based on both mutation data and gene expression levels (89,90). A similar approach could be implemented using NetCore, while including even more types of data, such as methylation, in order to identify novel cancer genes and modules. Finally, NetCore could be further used for other phenotype–genotype associations, based on various types of data, and it could also serve as an initial step for re-ranking of genes and extracting relevant features in the context of machine learning.

## DATA AVAILABILITY

All of the resources and software that have been used for the analysis are detailed in Table 2. NetCore is implemented in Python3 and is available for download via https://github.molgen.mpg.de/barel/NetCore.

## Supplementary Material

gkaa639_Supplemental_File
