# Supplementary material for: NetCore: a network propagation approach using node coreness
Source: Nucleic Acids Res. 2020 Jul 31;48(17):e98. doi: 10.1093/nar/gkaa639 (PMC7515737; doi:10.1093/nar/gkaa639)
Supplement: gkaa639_Supplemental_File [file gkaa639_supplemental_file.pdf]

NetCore: a network propagation approach using node coreness

Gal Barel[1] and Ralf Herwig[1*]

[1]Department of Computational Molecular Biology, Max-Planck-Institute for Molecular Genetics, Ihnestr. 63 – 73, 14195, Berlin, Germany

* To whom correspondence should be addressed. Tel: (030) 8413 1587 Email: herwig@molgen.mpg.de

Supplementary Methods

ConsensusPathDB high confidence network

The underlying PPI network for this study was constructed from PPIs collected from 19 different publicly available databases (see http://consensuspathdb.org for a list of databases). In order to improve false positive rates we have developed a confidence assessment for every interaction based on topological- and annotation-based measures [1] and assigned every interaction a score between 0 (low confidence) and 1 (high confidence).

For this study we kept interactions with confidence score > 0.95 what resulted in a PPI network consisting of 10,707 proteins and 114,516 interactions. This network falls into a large connected component as well as several smaller connected components ranging from sizes of 2-4. Since convergence of network propagation assumes that the underlying graph is connected we performed all analyses for this study on the largest connected component of the PPI network consisting of 10,586 proteins and 114,341 interactions.

To characterize the PPI network further we have conducted network analysis using the NetworkAnalyzer [2] plugin for Cytoscape [3].

Topological measures

Nodes: 10,586

Edges: 114,341

Network density: 0.002

Network clustering coefficient: 0.162

Network heterogeneity: 1.899

Node degree distribution

The PPI network shows the characteristic node degree distribution of biological networks with a couple of nodes with very high degree and most nodes having a smaller degree (Fig. 1). The power law fit has an R-squared of 0.903.

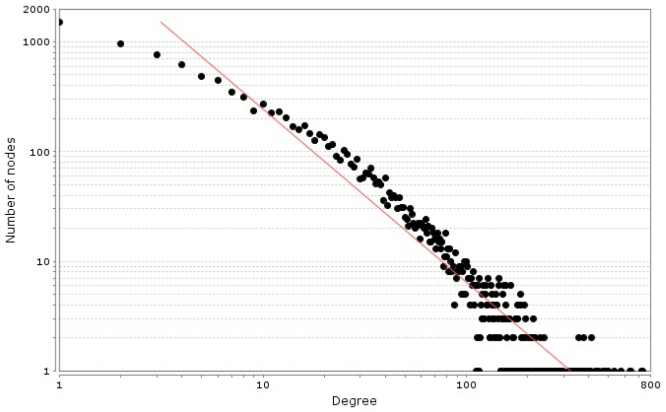

Supplementary Methods Figure M1. Node degree distribution in the ConsensusPathDB high confidence PPI network. Red line is a power law fit $y = ax^b$ with a = 9,193.4 and b = -1.577.

Shortest paths

The average shortest path length in the PPI network is 3.577, the longest distance of two nodes (i.e. the network diameter) is 11.

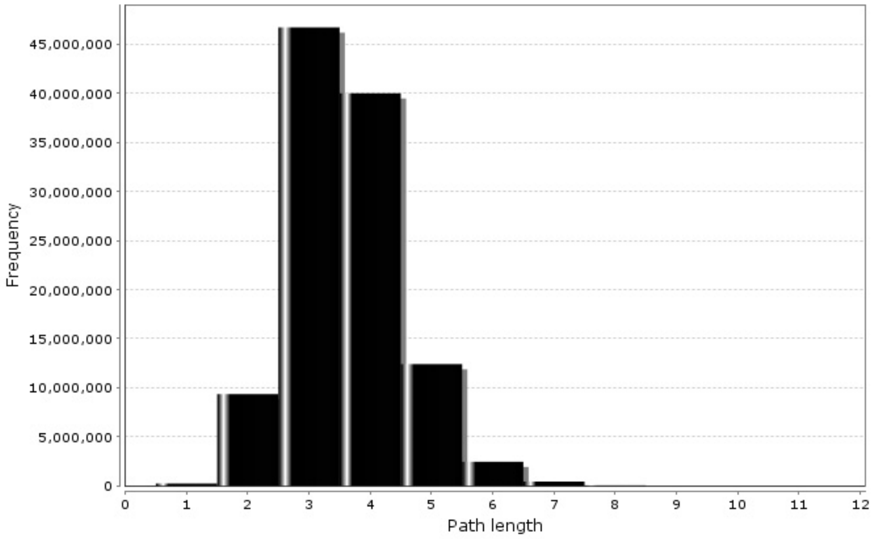

Supplementary Methods Figure M2. Histogram of shortest path lengths in the PPI.

Major hubs

Supplementary Methods Table M1 shows the 30 major hubs in the network.

Node	Node degree (max = 737)	Node core (max = 58)	Network of Cancer Genes (version 6.0)	11 GWAS Catalog gene-sets
TP53	737	51	Yes	
XPO1	726	42	Yes	
CUL3	645	58	Yes	Schizophrenia
UBC	642	38		
EGFR	574	38	Yes	
NTRK1	538	38	Yes	
GRB2	500	43	Yes	
RNF2	485	47		
CDK2	483	53		Rheumatoid arthritis, Vitiligo
MCM2	479	56		
ESR1	472	51	Yes	Breast Cancer, Height
CUL1	470	58	Yes	
HDAC1	445	38		
EP300	438	38	Yes	Crohn's disease, Schizophrenia
COPS5	431	58		
NPM1	413	58	Yes	
SIRT7	412	42		
APP	407	38		
MYC	405	38	Yes	Breast Cancer, Prostate Cancer, Height
YWHAZ	400	38		
EED	386	42	Yes	
CSNK2A1	382	38	Yes	
BRCA1	378	38	Yes	
CDC5L	378	38		
CUL7	374	46	Yes	
SNW1	372	38		
TRAF6	366	38	Yes	
HNRNPA1	358	42	Yes	
HNRNPU	354	58	Yes	
HSP90AB1	350	38		

Example of the network propagation process

To exemplify the process of network propagation we've chosen one of the modules that were identified by NetCore for Type-2 Diabetes. The module consists of seven genes, three of them were previously associated with the disease in the GWAS catalog: *ATP8B2*, *MTNR1B* and *PTPRD*. These nodes were scored with a weight of 1, and the rest of the nodes in the module with a weight of 0. Figure 3 displays the spread of the weights during the random walk with restart procedure for the sub-network of the seven nodes and their connections in the PPI network. After six steps the weights are already very close to the value at convergence. The weights for every node at every step are given by the table. We note that the nodes are also connected to other nodes in the network (which are not displayed here) and therefore their final weight is also affected by other connections. Since the restart parameter was set to 0.8, the weight that is propagated from the three disease nodes is only 0.2. Since we applied core normalization, the weight is spread according to the core of the neighbors. Finally, four more nodes are included in the sub-network, which had a significant P-value for their weight after the propagation, as well as a higher weight than the minimal that was chosen for the entire network ($w_{min} = 0.002$). *IL2RG* is connected only to *PTPRD*, while *MTNR1B* is connected to both *PTPRD* and *MTNR1A*. *LPAR6* is connected to both *PTPRD* and *ATP8B2*, which are not directly connected to each other, as well as to *MCOLN3*. Overall, our analysis was able to predict four novel genes that are connected to well-known disease genes and could be contributing to the disease phenotype.

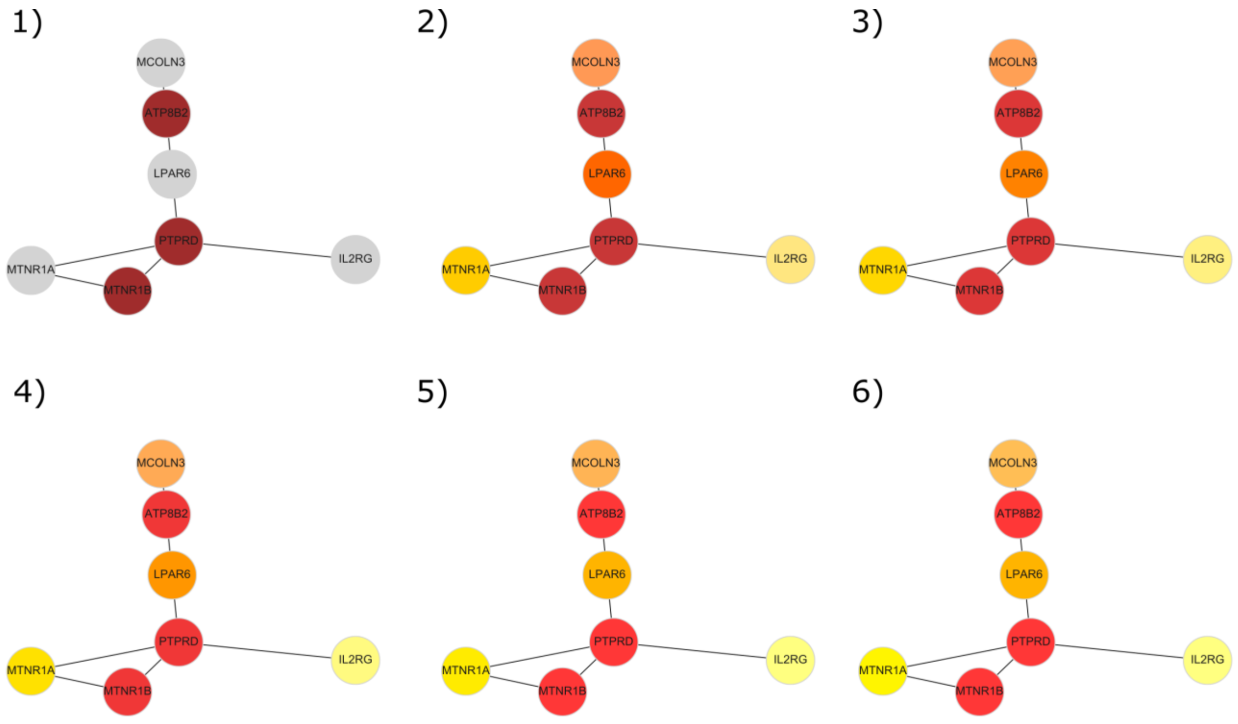

Gene	Step 1	Step 2	Step 3	Step 4	Step 5	Step 6	Final weights
ATP8B2	1	0.8	0.803708	0.802967	0.803017	0.803007	0.803007
IL2RG	0	0.02963	0.023806	0.023907	0.023886	0.023886	0.023886
LPAR6	0	0.109428	0.087593	0.088168	0.088052	0.088058	0.088057
MCOLN3	0	0.090909	0.07273	0.073479	0.07333	0.073339	0.073337
MTNR1A	0	0.055213	0.045794	0.045785	0.045741	0.045741	0.04574
MTNR1B	1	0.848148	0.841359	0.841104	0.841063	0.841062	0.841061
PTPRD	1	0.802717	0.80474	0.804245	0.804255	0.804252	0.804252

Supplementary Methods Figure M3. Example of network propagation in a network of seven genes. Only three of the genes are scored with 1 at the beginning of the propagation. The color of the node indicates its score, with red being the highest and yellow the lowest. The table summarizes the weights for each node in every one of the six propagation steps, as well as the final weight (at convergence).

References

[1] Kamburov et al. (2012) Nucleic Acids Res 40:W140-146.

[2] Assenov et al. (2008) Bioinformatics 24:282-284.

[3] Cline et al. (2007) Nat Protoc 2:2366-2382.

Supplementary figures

Figure S1

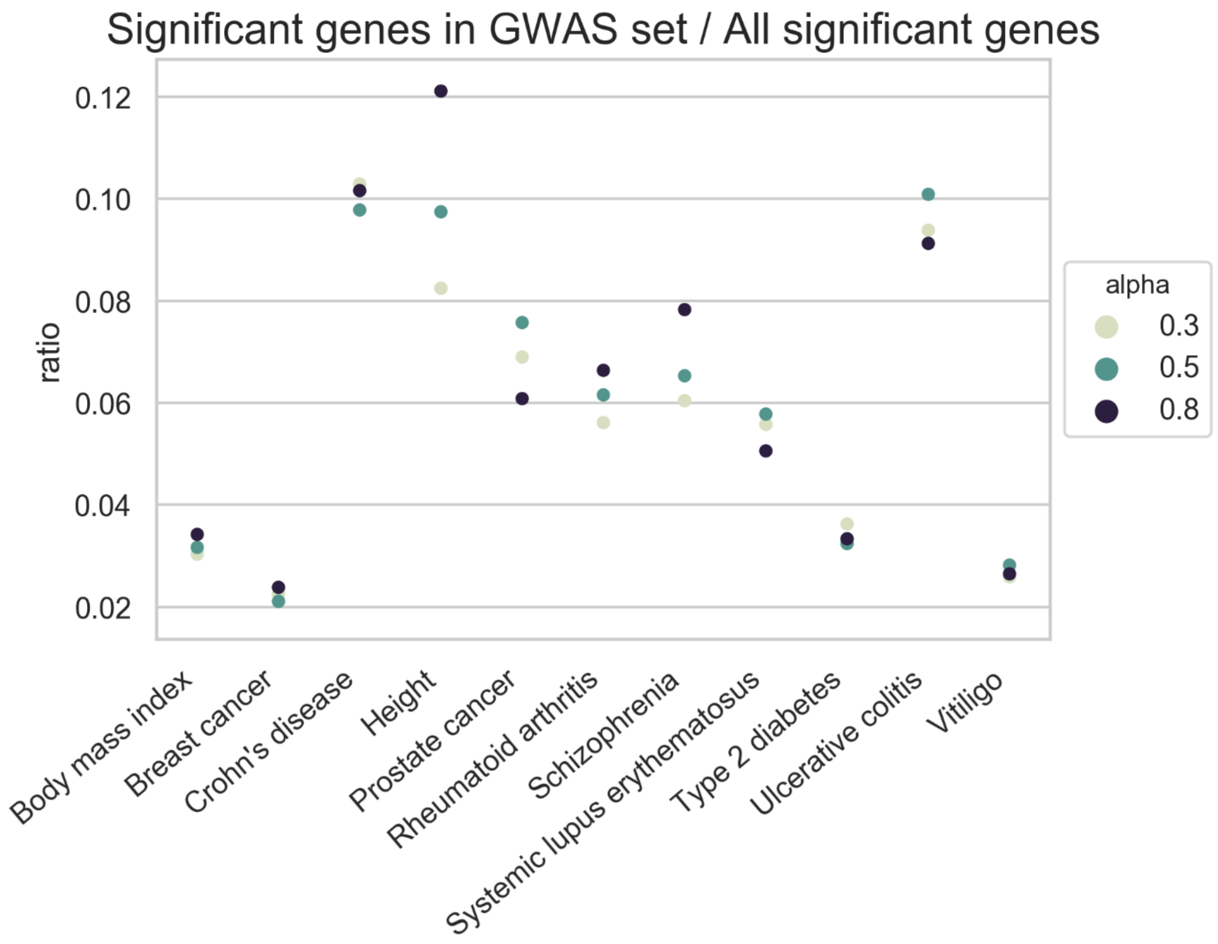

Figure S1: We compared the performance of NetCore in identifying 11 GWAS gene sets for three different values of the restart parameter α: 0.3, 0.5 and 0.8. The lower the value, the smaller the restart probability, which results in more of the weight being diffused throughout the network. We calculated the ratio between the number of significant genes that were reported by NetCore which belong to the input GWAS gene set, and the total number of significant genes that were reported by NetCore. In 5 of the 11 GWAS gene sets the highest performance is when α=0.8.

Figure S2

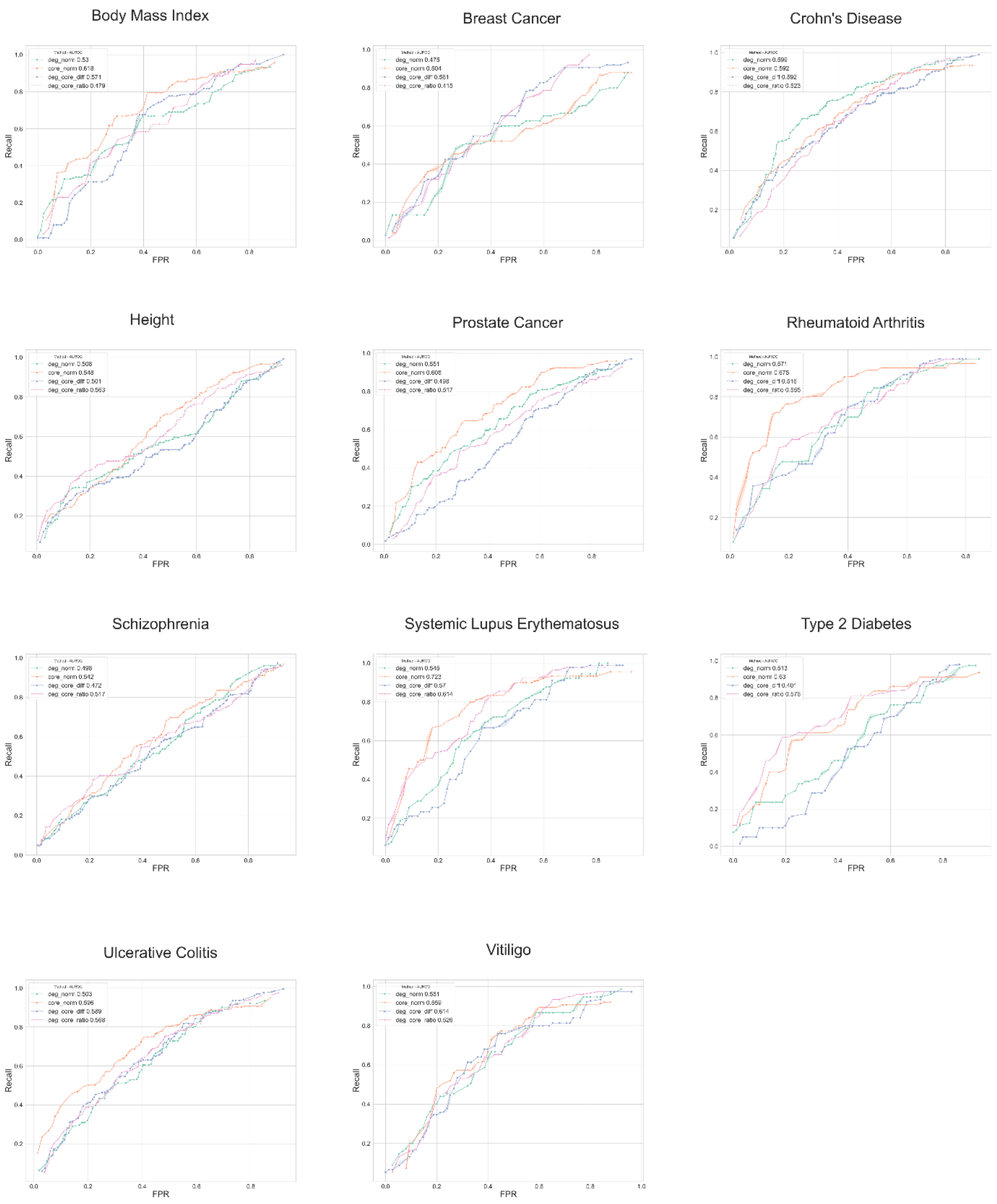

Figure S2: ROC curves for 11 GWAS gene sets. The different colors imply different normalization schemes. The lines depict the mean curve for the 5-cross validation results.

Figure S3

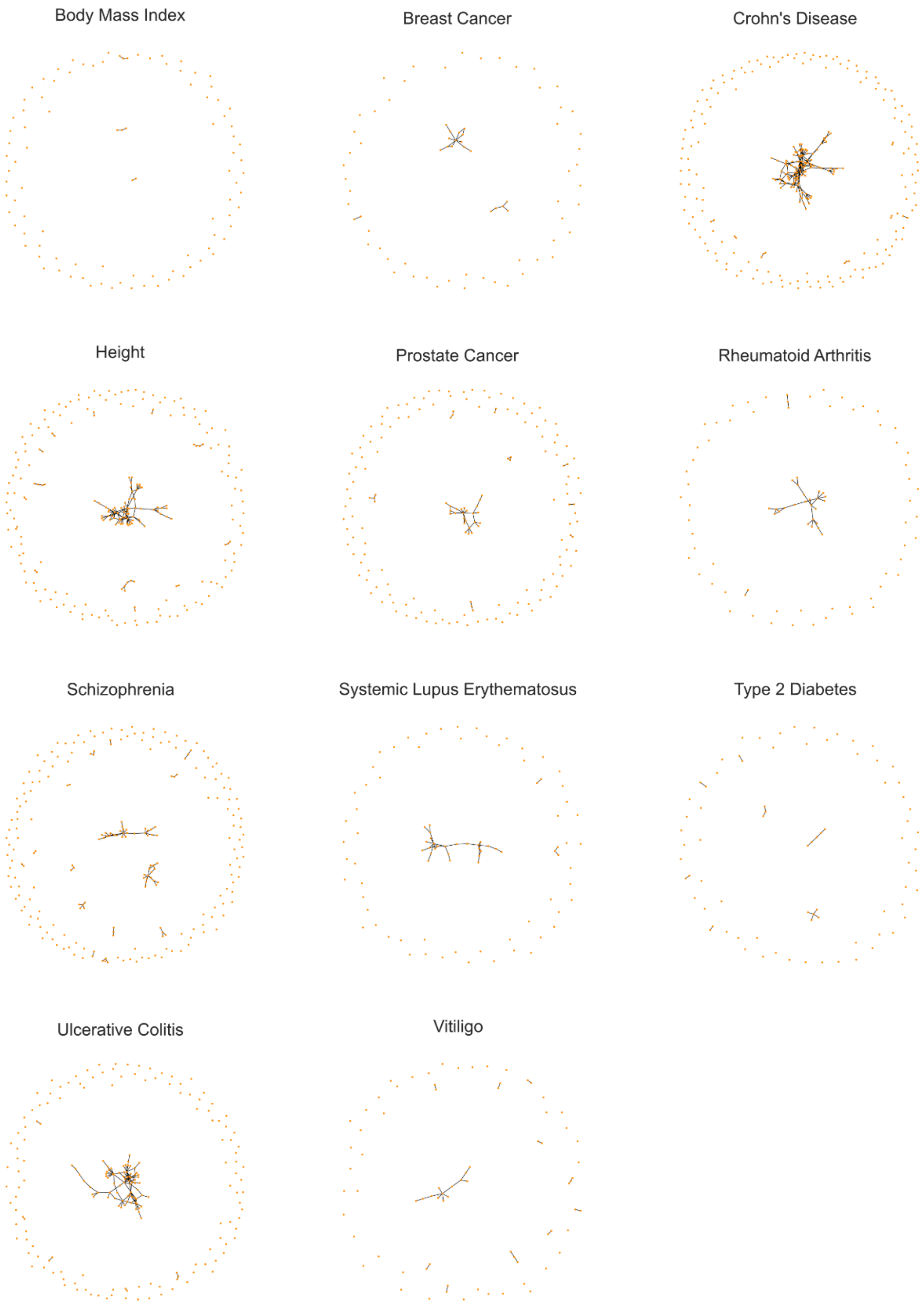

Figure S3: Seed sub-networks for 11 GWAS gene sets. Each node is a gene from the gene set, and each edge is an interaction from the PPI network.

Figure S4

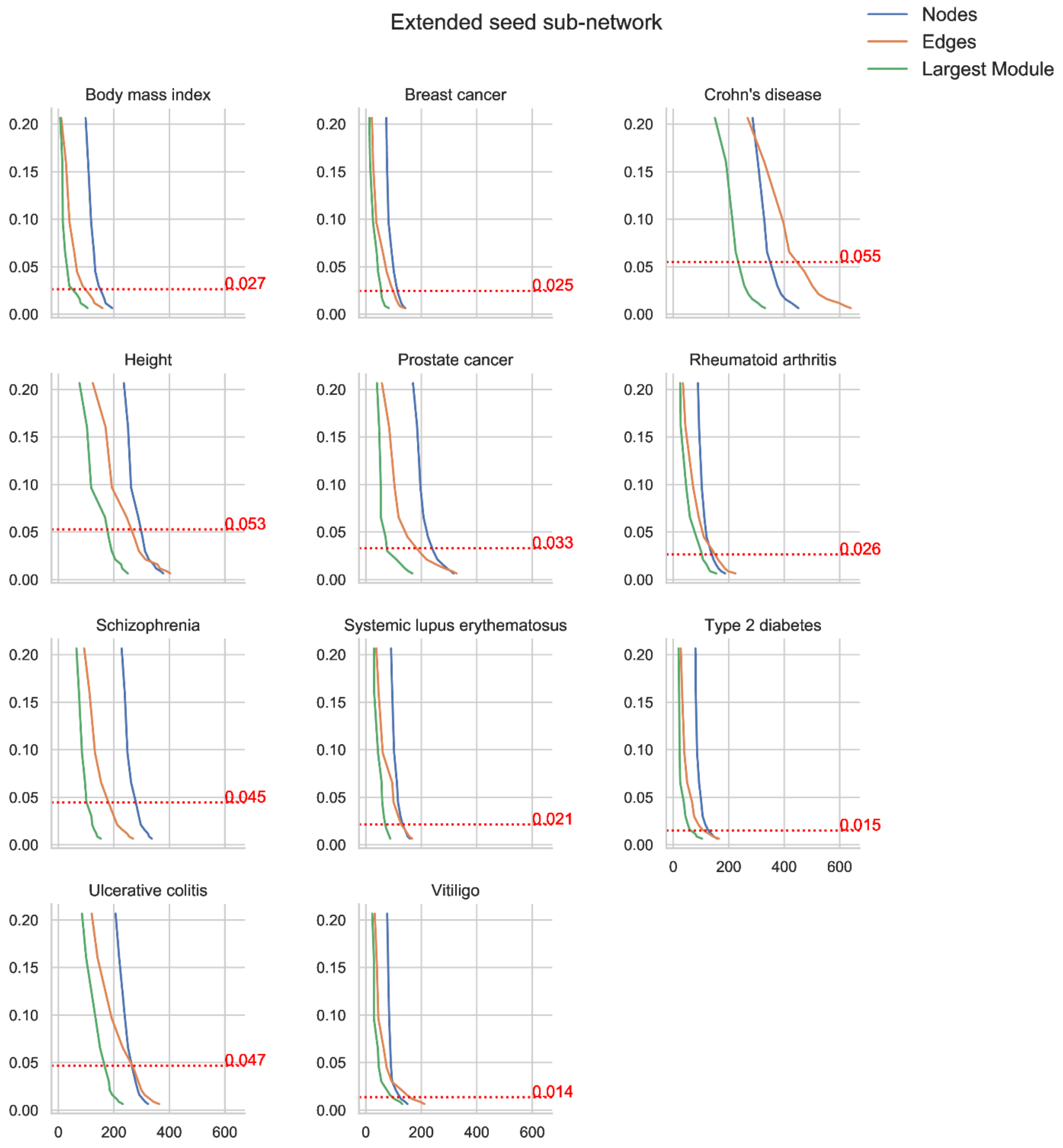

Figure S4: The effect of the weight threshold was examined when applying NetCore to 11 GWAS gene sets. The effect was measured, in the extended seed sub-network, by the number of nodes (blue), number of edges (orange) and number of nodes in the largest module (green). Y-axis is the weight after propagation, X-axis is the measured size. For each gene set the chosen weight threshold is marked (dashed red line), which was calculated by the 75th percentile of the weights after propagation, among the significant ($p<0.01$) nodes which are not in the input seed list (see Materials and Methods).

Figure S5

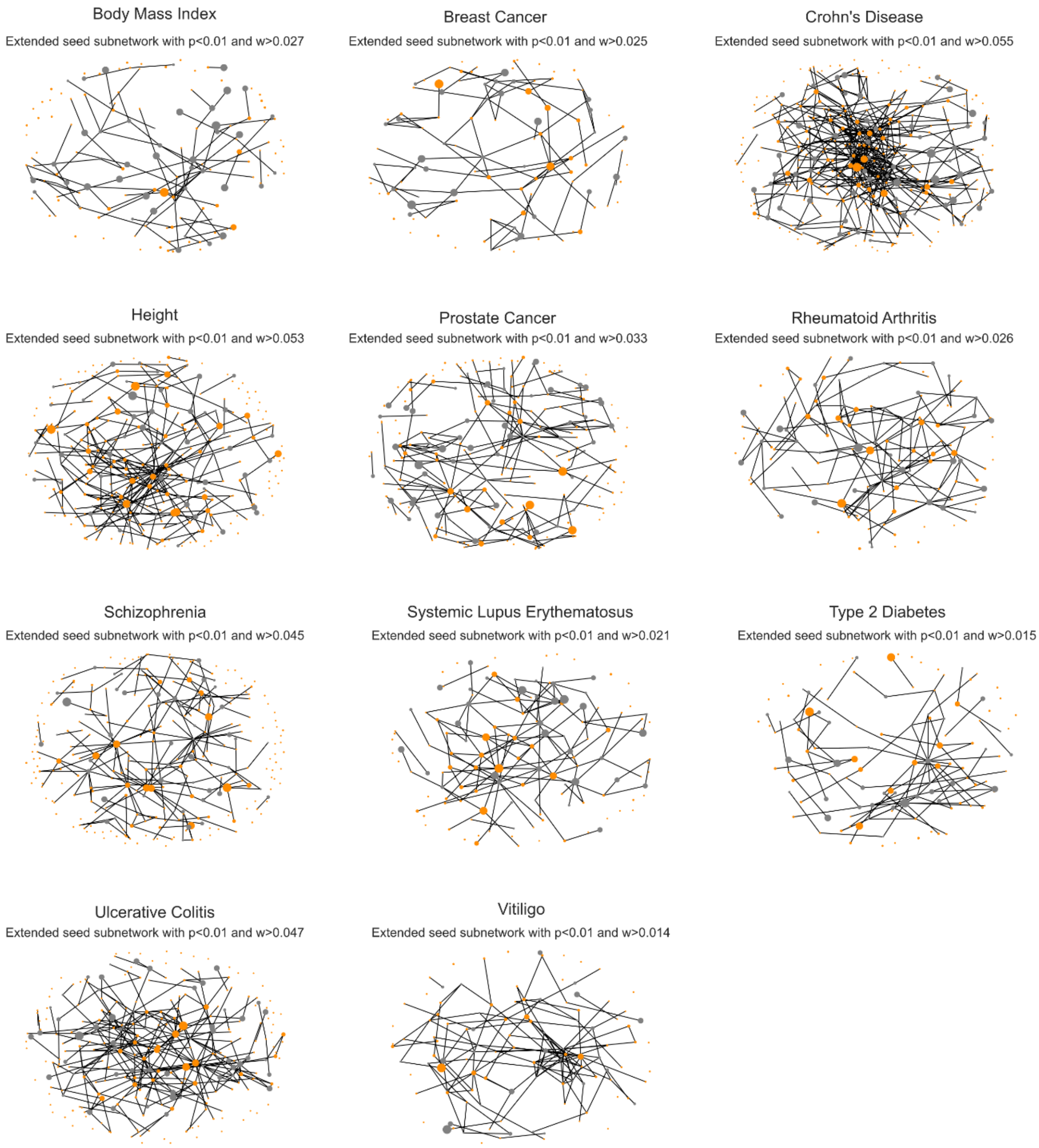

Figure S5: Extended seed sub-networks for 11 GWAS gene sets. The orange nodes are original seed nodes, the gray nodes were added to the seed sub-network after the propagation, according to their results (significant P-value of $p < 0.01$ and a minimum weight, which is calculated based on the weights distribution after the propagation). The sizes of the nodes reflect their weights after the propagation. The edges are originally from the PPI network.

Figure S6

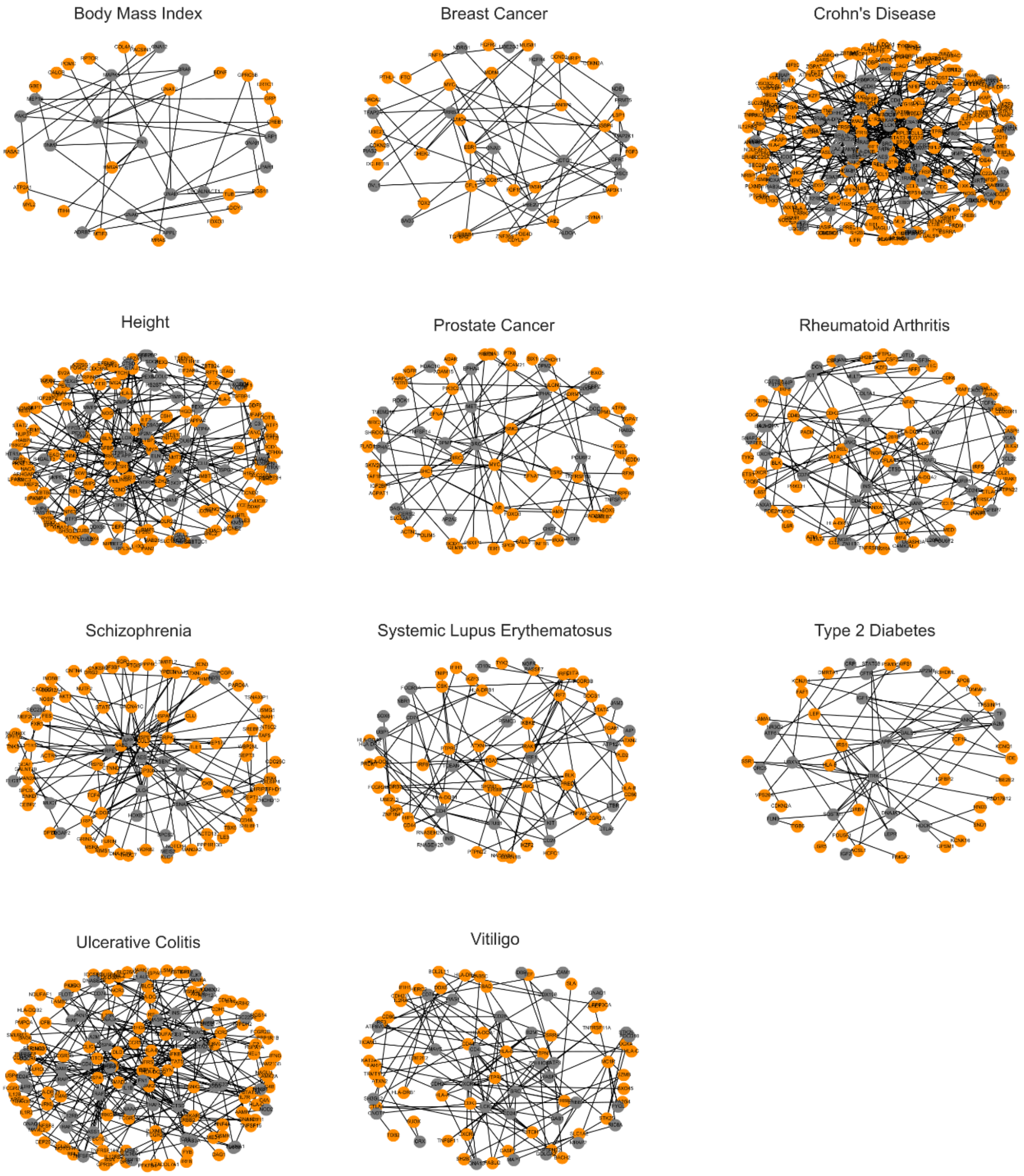

Figure S6: Largest modules for 11 GWAS sets based on NetCore. The largest module is extracted from the extended seed sub-network, where it is the largest connected component of the sub-network. Orange genes are in the original gene sets (seed nodes), and gray ones were added after the propagation. The edges are from the PPI network.

Figure S7

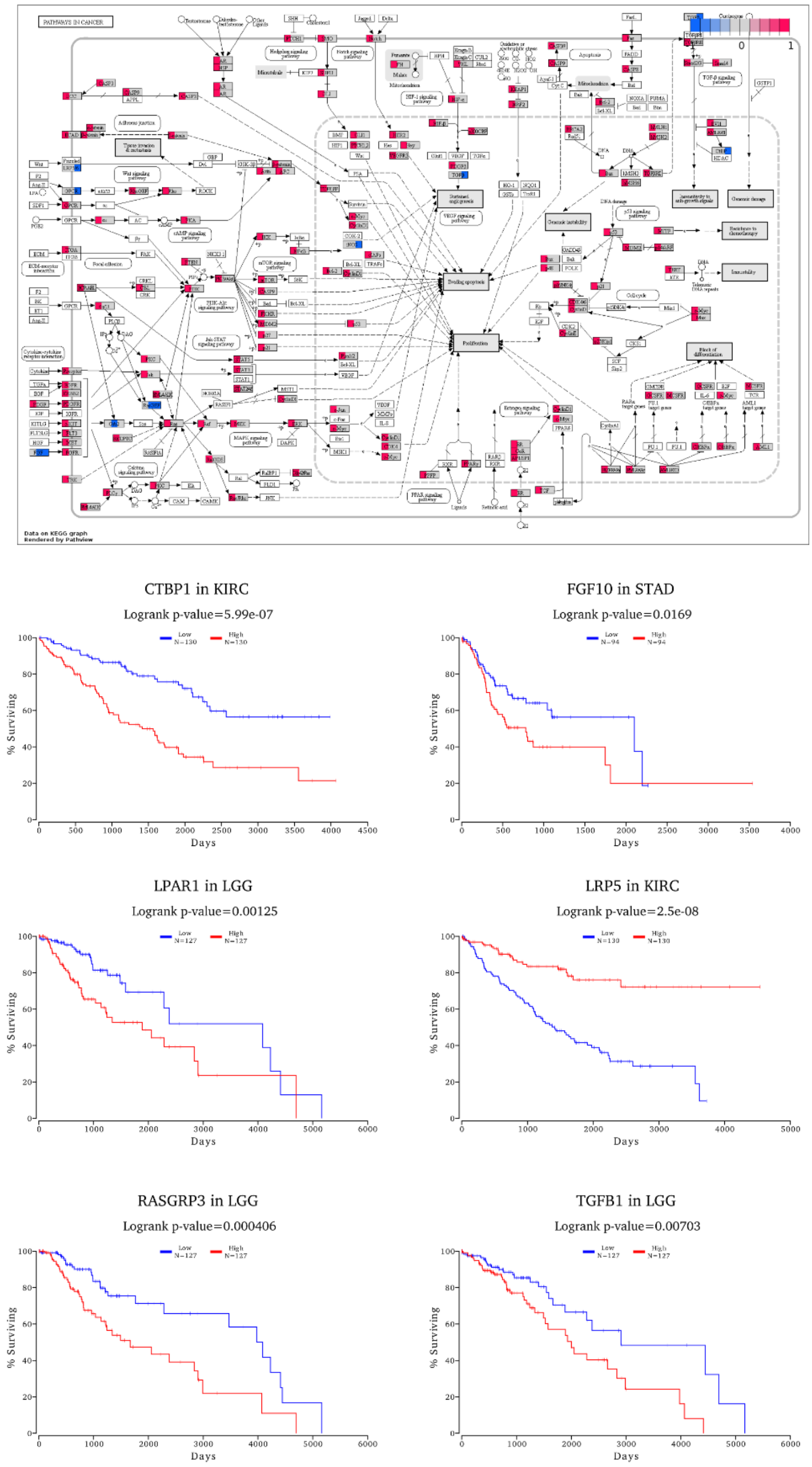

Figure S7: "Pathways in Cancer". The pathway as depicted by KEGG and generated using Pathview (1). The colored nodes are present in the module from NetCore. Red nodes are present in the NCG cancer consensus list. Blue nodes are newly predicted genes, some are present in the NCG cancer candidate list, and some not. The Cox regression plots are based on

TCGA survival data for six genes in the pathway that were predicted by the module and are not present in either the consensus or candidate cancer lists. The plots were generated using the *OncoLnc* web service. The results are shown for the cancer type with the lowest FDR-corrected P-value. The cancer abbreviations are according to the TCGA project. *LGG* refers to Brain Lower Grade Glioma. *KIRC* refers to Kidney renal clear cell carcinoma. *STAD* refers to Stomach adenocarcinoma.

1. Luo, W., Pant, G., Bhavnasi, Y.K., Blanchard, S.G., Jr. and Brouwer, C. (2017) Pathview Web: user friendly pathway visualization and data integration. *Nucleic acids research*, **45**, W501-w508.
